# Permissiveness and competition within and between *Neurospora crassa* syncytia

**DOI:** 10.1093/genetics/iyad112

**Published:** 2023-06-14

**Authors:** Alexander P Mela, N Louise Glass

**Affiliations:** The Plant and Microbial Biology Department, University of California Berkeley, Berkeley, CA 94720, USA; The Plant and Microbial Biology Department, University of California Berkeley, Berkeley, CA 94720, USA; The Environmental Genomics and Systems Biology Division, The Lawrence Berkeley National Laboratory, Berkeley, CA 94720, USA

**Keywords:** syncytia, *Neurospora crassa*, nuclear competition, heterokaryon incompatibility, allorecognition, cell fusion

## Abstract

A multinucleate syncytium is a common growth form in filamentous fungi. Comprehensive functions of the syncytial state remain unknown, but it likely allows for a wide range of adaptations to enable filamentous fungi to coordinate growth, reproduction, responses to the environment, and to distribute nuclear and cytoplasmic elements across a colony. Indeed, the underlying mechanistic details of how syncytia regulate cellular and molecular processes spatiotemporally across a colony are largely unexplored. Here, we implemented a strategy to analyze the relative fitness of different nuclear populations in syncytia of *Neurospora crassa*, including nuclei with loss-of-function mutations in essential genes, based on production of multinucleate asexual spores using flow cytometry of pairings between strains with differentially fluorescently tagged nuclear histones. The distribution of homokaryotic and heterokaryotic asexual spores in pairings was assessed between different auxotrophic and morphological mutants, as well as with strains that were defective in somatic cell fusion or were heterokaryon incompatible. Mutant nuclei were compartmentalized into both homokaryotic and heterokaryotic asexual spores, a type of bet hedging for maintenance and evolution of mutational events, despite disadvantages to the syncytium. However, in pairings between strains that were blocked in somatic cell fusion or were heterokaryon incompatible, we observed a “winner-takes-all” phenotype, where asexual spores originating from paired strains were predominantly one genotype. These data indicate that syncytial fungal cells are permissive and tolerate a wide array of nuclear functionality, but that cells/colonies that are unable to cooperate via syncytia formation actively compete for resources.

## Introduction

In many filamentous ascomycete fungi, the main vegetative mycelial growth form is a haploid, multinucleate, interconnected syncytium formed by somatic cell fusion. Somatic fusion between germinated asexual spores (germlings) and hyphae can allow genetically distinct nuclei to occupy the same cytoplasm, a so-called heterokaryon (Supplementary Fig. 1). Syncytia occur across multiple domains of life, including muscle cells, the placenta in mammals, plasmodia of slime molds, and heterokaryotic cells of the angiosperm *Utricularia* (Mela *et al.* 2020). A potential advantage of syncytia in filamentous fungi is that nuclei and organelles have an opportunity to exchange resources (‘public goods’) and potentially complement deleterious mutations either through parasexual mitotic recombination or by being in a shared cytoplasm (Pontecorvo 1956; Anderson *et al.* 2018). Heterokaryons in filamentous fungi have been postulated to provide advantages of diploid cells, where genetically different haploid nuclei can coexist in a common cytoplasm. Further studies showed that regional differences in spatial arrangement of nuclei and mitotic synchronicity (Rosenberger and Kessel 1967; Clutterbuck 1970; Freitag *et al.* 2004), as well as transcription rates and nuclear number, can facilitate genotypic and phenotypic plasticity within syncytia (Schuurs *et al.* 1998; Gladfelter 2006; Gladfelter and Berman 2009). However, a disadvantage of syncytial growth strategies is that organelles (e.g. nuclei or mitochondria) can become non-cooperating entities, or “cheaters’, within a colony, which benefit from intracellular or extracellular resources (public goods) produced by the “cooperator”, but that do not share the cost of producing said resources (Smith and Schuster 2019). Some examples of such public goods in filamentous fungi could be secreted enzymes used for plant cell wall deconstruction, intracellular metabolites, antibiotic production, and compounds associated with the production of spores and tissues for asexual/sexual reproduction. Non-cooperators can also potentially perpetuate deleterious mutations, thereby posing a detriment to the colony overall. Such competition can lead to the entire colony being overrepresented by a mutant genotype, as is the case with the *Neurospora crassa* “poky” mutant. The poky mutant contains mutations in its mtDNA (Mannella and Lambowitz 1978; Akins and Lambowitz 1984) that provide the mutant with a higher relative fitness in syncytia due to increased replication rate of mitochondria, despite a range of deleterious pleiotropic phenotypes, most notably a defective growth rate, the presence of aberrant mitochondrial ribosomes, and cytochrome deficiencies. Competition can, in some cases, even result in the breakdown of the syncytium into homokaryons (Mannella and Lambowitz 1978; Rayner *et al.* 1995; Rayner 1996). An evolutionary study by Bastiaans *et al.* (2016) in *N. crassa* and subsequent genotyping of the associated evolutionary lines by Grum-Grzhimaylo *et al.* (2021) showed that syncytia carried across multiple generations resulted in the consistent recovery of mutants blocked in somatic cell fusion, specifically mutations in *so* (*soft*), *ham-2*, and *ham-8* (Xiang *et al.* 2002; Fleissner *et al.* 2005; Fleissner and Glass 2007; Dettmann *et al.* 2013; Fu *et al.* 2014). The evolved syncytia bearing the fusion mutant nuclei did not exhibit the same growth disadvantages as homokaryotic fusion mutants in monoculture, suggesting that they indeed benefit from excreted and/or leaked public goods. Multicellular organisms can limit the spread of cheaters by the transition to a unicellular, uninucleate state during sexual reproduction, which creates a bottleneck of defective mutations or selfish elements, by reallocating them between haploid progeny (Grosberg and Strathmann 2007).

In *N. crassa*, there are at least three checkpoints that regulate somatic cell fusion. The first checkpoint regulates chemotropic interactions, the second checkpoint regulates cell wall dissolution upon contact, and the third checkpoint regulates the establishment of a heterokaryon following somatic cell fusion (Gonçalves et al. 2020). In the third checkpoint, if cells have different allelic specificity at *vegetative incompatibility* or *heterokaryon incompatibility* (HI) *het* loci, the fusion compartment is compartmentalized and undergoes a rapid regulated cell death process (Glass and Dementhon 2006; Daskalov *et al.* 2017; Goncalves *et al.* 2017). In *N. crassa*, heterozygosity at *het* loci has been shown to reduce heterokaryons found in nature (Pandit and Maheshwari 1996). In *N. crassa*, the mating type locus functions to trigger regulated cell death following somatic cell fusion of *mat a* and *mat A* cells (Pittenger 1957; Griffiths 1982; Glass *et al.* 1990; Glass and Kuldau 1992). Specific mutations in *mat a-1* or *mat A-1* abolish mating type incompatibility, but not sexual compatibility, while extragenic suppressor mutations at the *tol* (tolerant) locus suppress mating type incompatibility (Newmeyer 1970; Griffiths 1982; Jacobson 1992; Saupe *et al.* 1996; Shiu and Glass 1999). Some proteins encoded by *het* loci have functional and sequence homology to proteins involved in innate immunity in animals (Dyrka *et al.* 2014; Daskalov *et al.* 2016, 2020; Dyrka *et al.* 2020; Daskalov and Glass 2022); HI reduces the transmission of mycoviruses and the transmission of defective genetic elements, including mitochondria associated with senescence (Debets *et al.* 1994; Liu and Milgroom 1996; van Diepeningen *et al.* 1997; Debets and Griffiths 1998).

In filamentous fungi, the production of asexual spores (conidia) is used as a measure of fitness (Pringle and Taylor 2002; Gilchrist *et al.* 2006). In Aspergilli *spp*., the formation of conidiophores is associated with recruitment of nuclei from basal hyphae, which undergo synchronous mitotic divisions to form uninucleate, haploid asexual spores (conidia) (Mims *et al.* 1988; Adams *et al.* 1998; Park and Yu 2012). However, in *N. crassa*, conidiophores from aerial hyphae undergo minor and major cell wall constrictions, compartmentalizing nuclei into spores via septation to produce multinucleate, haploid conidia (Matsuyama *et al.* 1974; Cole 1986; Springer and Yanofsky 1989; Ruger-Herreros and Corrochano 2020) (Supplementary Fig. 1), reflective of nuclear populations within the multinucleate syncytia.

In this study, we examined the dynamic mixing of differentially tagged histone H1 nuclei that differed in auxotrophic markers, intracellular public goods, or heterokaryon incompatibility factors. The relative fitness of each nuclear genotype was analyzed in a large population of asexual spores derived from syncytia via flow cytometry and fluorescence microscopy. Here, we show that the syncytia of *N. crassa* are permissive toward the propagation of nuclei with auxotrophic requirements or nuclear spacing defects in mycelia and asexual spores, despite the clear disadvantage of producing such asexual progeny. However, allorecognition resulting in cell death and the inability to undergo somatic cell fusion was a sufficient selective pressure to increase fitness of one nuclear genotype over another, resulting in a “winner-takes-all” phenotype, where one nuclear genotype reached near saturation in the harvested population of asexual spores. These results illustrate both the cooperative and competitive nature of fungal syncytia.

## Materials and methods

### Strains, media, culturing methods, and sexual crosses

All *N. crassa* strains used in the study are listed in Supplementary Table 1. The wild-type *N. crassa* genetic background for tagged and crossed strains were OR74A (FGSC 2489 or FGSC 4200) (Colot *et al.* 2006). All deletion strains used in this study were obtained from the Fungal Genetics Stock Center (http://www.fgsc.net/) single-gene *Neurospora* deletion collection (McCluskey 2003; Colot *et al.* 2006). NCU numbers for the loci in this study are as follows: *his-3* NCU04393; *csr-1* NCU00726; *arg-5* NCU05410; *arg-12* NCU01667; *ro-3* NCU03483; *ro-10* NCU10696; *tol* NCU03378; *cwr-1* NCU03180; *cwr-2* NCU03182; *rcd-1* NCU05712; *so* NCU02794; and *fl* NCU08726. Culturing and crossing, using Vogel's media (Vogel 1964) and Westergaard's media (Westergaard *et al*. 1947), respectively, were performed as previously described, with modification (Davis and De Serres 1970; Perkins 1986). All deletion strains from single-gene deletion collection were backcrossed once to either Wild Type (WT), histone H1 (H1)-eGFP or WT, H1-mCherry strains listed in Supplementary Table 1, to obtain histone-tagged homokaryons; the genotype of progeny was verified by PCR or phenotypic characterization. *Fluffy mat A* and *fluffy mat a* strains (BF and A7, respectively, Supplementary Table 1) are routinely crossed to *mat A* strains (FGSC 2489) to maintain fertility and which were used to determine mating type of progeny. Auxotrophic supplements were prepared in 100× and 1,000× stock solutions of 60-mg/mL L-arginine (Sigma-Aldrich) and 10-mg/mL calcium pantothenate (Fisher Biotech), respectively, filter sterilized, and stored at 4°C. Supplements were added to autoclaved media precooled to ∼5°C before pouring in plates or race tubes. Crosses were performed on Westergaard's medium (Westergaard *et al*. 1947). Ascospores were activated by incubating in PCR tubes at 6°C for 30 minutes in a Thermo Fisher Scientific MiniAmp thermal cycler. Ascospores were individually excised from plates onto slants containing 1-mL media (with supplements as needed), and progeny were subsequently verified by PCR for the proper genotype, tested for mating type, auxotrophic mutation and for fluorescence (Perkins 1986). Primers used in this study are listed in Supplementary Table 2. Strains used in this study are available at the Fungal Genetics Stock Center (https://www.fgsc.net/); FGSC numbers are listed in Supplementary Table 1.

### Cloning and transformation

Spheroplast preparation and transformation by electroporation were performed as previously described (Schweizer *et al.* 1981; Vollmer and Yanofsky 1986), except for selecting for resistance to hygromycin B (250 units/mL; Calbiochem). The H1-mCherry-tagged parent strain was constructed with a modified pMF272 *his-3* targeting vector (Pccg-1-histone H1-5x Gly linker-eGFP or mCherry-Tccg-1), (Freitag *et al.* 2004). The eGFP sequence was removed from pMF272 by restriction digestion in universal buffer at 37°C with NEB *Xba*I and *Eco*RI restriction enzymes. An ∼1,174-bp product containing the native histone H1 sequence, along with a 5x Gly linker sequence was amplified from FGSC WT 2489 gDNA (Supplementary Table 2). A ∼1,083-bp product containing the mCherry gene and Tccg1 terminator sequence was amplified from plasmid pNLY1-4. Using Q5 NEB Polymerase, fusion PCR was conducted to combine fragments, followed by gel purification using a Qiaquick Qiagen Gel Extraction Kit, following the manufacturer's instructions. Fragments were cloned into digested vector pMF272 between the Pccg1 promoter and downstream his-3 flanking region, using NEB HiFi DNA Assembly Cloning Kit following manufacturer's instructions.

**Fig. 1.**
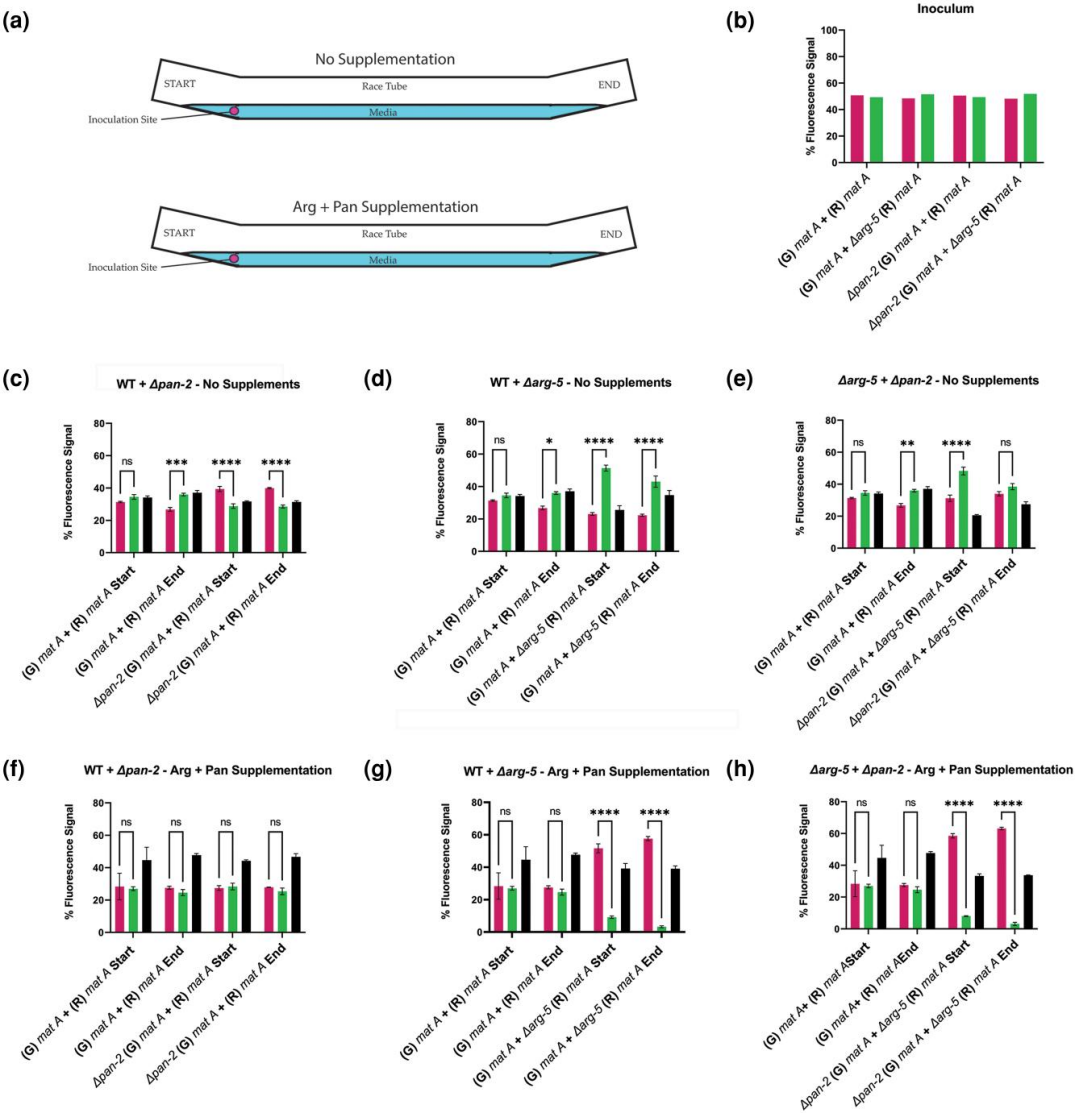
Flow cytometry analysis of prototrophic and auxotrophic spore pairings with and without supplementation. a) Schematic of race tubes labeling the media (blue) with no supplementation or arginine + pantothenic acid supplementation, along with the inoculation site (magenta circle). b) Inoculum ratios of spore pairings used in this race tube experiment. c) *his-3::hH1-mCherry* (R) *mat A* + *Δpan-2 his-3::hH1-eGFP* (G) *mat A* (74DM + 11U). d) *mat A* (G) + *Δarg-5* (R) *mat A* (10BI + 102CC). e) *Δarg-5* (R) *mat A* + *Δpan-2* (G) *mat A* (102CC + 11U) spore pairings inoculated at approximately equal ratios and grown in race tubes for 7 dpi at 3°C with no media supplementation; or f–h) with arginine + pantothenic acid supplementation. Flow cytometry was conducted to analyze the relative percentage of each fluorescently tagged nuclear genotype in asexual spore populations derived from syncytia. Spore pairings in each biological replicate were derived from a single inoculum sample, and the ratios of each partner in the inoculum are shown in the “Inoculum” graph (panel b). “Start” denotes that spores were collected from the opening of the race tube most proximal to the inoculation site on day 7, and “End” denotes that the spore sample was collected from the portion of the race tube most distal from the site of the inoculation on day 7. Left bars (magenta) in graphs denote fluorescence signal from homokaryotic spores with the presence of only histone H1-mCherry-tagged nuclei, right (green) bars (panel b) or middle (green) bars (panels c-h) in graphs denote fluorescence signal from homokaryotic spores with the presence of only histone H1-eGFP-tagged nuclei, and black bars in graphs denote spores with signal from both histone H1-mCherry and histone H1-GFP nuclear tags in the same cell. Statistical analysis was conducted using two-way ANOVA followed by Tukey’s honestly significant difference (HSD) post hoc tests. *P < 0.05; **P < 0.01; ***P < 0.001; ****P < 0.0001; ns = not significant. *N* = 3. Error bars = SEM.

FGSC 6103 histidine auxotrophic conidia were harvested in 30-mL ice-cold 1 m sorbitol and filtered through 4 sheets of cheesecloth. Samples were centrifuged at 4°C and 3,400 rpm for 10 minutes, and the pellet was washed 3× with 15–30-mL ice-cold sorbitol. The conidial pellet was gently resuspended in as small volume of sorbitol, and spore concentration was counted and normalized to 5 × 10^9^ conidia/mL. The plasmid was linearized using NdeI/SspI restriction site, and transformations were carried out as previously described (Colot *et al.* 2006). Transformants were plated with precooled recovery media (1.4-mL FIGS (10X) + 14-mL top agar [VMM Nitrate Salts (1X) with 1% agar)] onto thin layer of bottom agar (VMM Nitrate Salts (1X) with 2% agar, FIGS (1X), and hygromycin), and incubated at 3°C for 2–3 days. Primary transformants were selected for histidine prototrophy and hygromycin resistance and analyzed by PCR for insertion at the proper locus, and nuclear fluorescence was verified by microscopy. A selection of transformants with correct genotype was backcrossed to FGSC 2489 strain to obtain homokaryons with desired genotype (Ebbole and Sach 1990).

### Microscopy

Each strain was harvested in ddH20, filtered through cheesecloth, and normalized to 1.5 × 10^7^ conidia/mL before plating. All microscopy was conducted on a Zeiss Axioskop 2 MOT fluorescence microscope equipped with a CoolLED pe-300 microscope illuminator. Imaging was conducted using a QImaging Cool 12-bit Mono digital camera equipped on the fluorescent microscope and scale bars were inserted in iVision imaging software. Micrographs were analyzed, and brightness/contrast changes were uniformly applied to entire image in ImageJ; figures were prepared in Adobe Illustrator 2022 v26.5.

### Methylene blue vital dye staining

For methylene blue (MB) experiments, strains were normalized to 1.5 × 10^7^ conidia/mL, mixed 1:1, and added to 10-mL Vogel's minimal liquid media with 2% sucrose in a small petri dish on top of a flame-sterilized glass coverslip. Petri dishes were incubated in dark at 3°C for 5–6 hpi, and media was removed/discarded and immediately replaced with staining solution. Methylene blue trihydrate (Sigma; Lot # 99H3618) stock solution was prepared fresh each experiment by adding 0.01 to 1-mL ddH20 in small Eppendorf tube, and diluted 200× for working solution in 10-mL sterile ddH20. The surface of the mycelium-side-up coverslip was covered with 3 mL of working solution in petri plate and allowed to incubate for 3 minutes. The dye was removed from the petri dish, and 3× subsequent washes were done with 5-mL ddH20 or until the stain was visibly removed. Coverslips were immediately mounted hyphae-side-down onto a clean glass microscope slide and viewed with a 20× objective lens. Within 20–30 minutes, brightfield images were taken to visualize the blue staining of dead/dying hyphal compartments and/or clear viable hyphal compartments, and red and green fluorescence channels were observed to verify the presence of nuclei from each partner of the germling fusion.

### Race tube experiments

Race tubes were 39 cm in length with a 1.2 cm internal diameter, and solidified media occupied approximately 30 cm across the bottom of each tube. Bird's Media was prepared fresh as previously described with 1.8% sucrose as the primary carbon source and 1.5% BTS Superpure Agar as the solidifying agent; 13 mL was aliquoted into sterile race tubes from the same media batch in each experiment (Metzenberg 2004). Strains were harvested in 2-mL ddH20 from slants and filtered through multiple layers of cheesecloth to remove mycelial fragments. Fresh spores were normalized to 1.95 × 10^7^ conidia and mixed in 1:1 ratio prior to inoculation in a 10-μL droplet at the “Start” of each race tube in at least triplicate. In spore pairings with *ropy* mutants, the initial inoculum was a 1:1.3 ratio (1.95 × 10^7^ conidia/mL:2.54 × 10^7^ conidia/mL, prototrophic:*ropy* spores), to normalize the number of *Δro-3* and *Δro-10* spores with detectable fluorescence signal. Inoculum ratios for each spore pairing were quantified by flow cytometry on day 0 of each independent experiment. Race tubes were placed in an incubator at 3°C in the dark O/N to ensure inoculum was dry, and initial growth had started before flipping race tubes to avoid potential pooling of CO_2_, which could potentially affect conidiation (Sargent and Kaltenborn 1972). Race tubes were incubated ∼7 days. To harvest the spores from race tubes, 1 mL of ddH20 was added to the “start” and “end” of each tube, and gently pipetted up and down to free spores into solution, followed by filtration through sterile cheesecloth. Race tubes and controls were harvested together and analyzed on the same day to ensure consistent results.

For linear growth rate determinations, conidia were harvested, filtered, normalized to 1.95 × 10^7^ conidia/mL, and inoculated in 10-μL droplets from the same inoculum in at least triplicate onto race tubes containing fully supplemented Bird Media, and incubated in the dark at 3°C. Mycelial growth was measured (mm) each day on race tubes at the same time for 3 days.

### Flow cytometry

Nuclear fluorescence within spores were recorded for ∼10,000 to ∼20,000 events for remaining samples on an LSR Fortessa Analyzer using PE-Texas Red 600LP | 610/20 filter and FITC 505LP | 525/50 filter. Voltages are calibrated with control samples of each individual strains used in the experiment. Events from the sample of interest are counted by the flow cytometer for each replicate sample per spore pairing. Cells are gated for spore size/granularity and the presence of cell stuck together (doublets) based on [side scatter area × forward scatter area] and [forward scatter height × forward scatter area] measurements, respectively, to avoid counting spores too large/small/granular, cell debris, and cells stuck together. Intial data showed that cytoplasmic GFP and cytoplasmic dsRed could be used to assess heterokaryotic spore formation, but the fluorescence signal was much brighter using histone H1-eGFP and histone H1-mCherry. The data were further grouped in a two-parameter density plot, based on fluorescence signal in 4 quadrants (Supplementary Fig. 2). The PE-Texas Red filter detects hH1-mCherry fluorescence, and FITC filter detects hH1-GFP fluorescence. Spore size, nuclear number, and diffusion of histones are not likely to affect the majority of the spores counted within each quadrant, although a fraction of spores recorded to be on the cusp between quadrants could be over/under counted as “mixed” spores, meaning that they are showing both GFP and RFP fluorescence signals. BDFACs Diva software was used to analyze samples, and the same experimental parameters were used for every flow cytometry experiment in this study, with manual adjustments to voltage for proper discrimination of single fluorophores between runs. Graphing and statistical tests for each experiment were conducted using GraphPad Prism 9.4.1.681. The statistical significance within each dataset was determined by running a two-way ANOVA with replication. Multiple comparisons test was done using parameters ensuring all cell means were compared regardless of rows and columns. Corrections for multiple comparisons were conducted using statistical hypothesis testing with Tukey’s pairwise post hoc t-tests (family-wise alpha threshold—0.05; 95% confidence interval; *N* = 3–6).

## Results

### Prototrophic vs auxotrophic nuclear cooperation in fungal syncytia

Within the fungal multinucleate syncytium, the number of nuclei within a hypha can vary, with the large trunk hyphae (∼10 μm in diameter) containing large numbers of nuclei, while other hyphae contain fewer numbers of nuclei (Turian and Bianchi 1972; Freitag *et al.* 2004). To assess nuclear composition of syncytia under different genetic scenarios in a *N. crassa*, we analyzed frequency of nuclear genotypes in asexual spores using flow cytometry. Asexual spores in *N. crassa* contain, on average, between 2 and 5 nuclei that are compartmentalized within a spore during conidiation (Turian and Bianchi 1972; Ruger-Herreros and Corrochano 2020) (Supplementary Fig. 1). To facilitate these experiments, we introduced a histone H1-GFP allele to mark nuclei of one strain and H1-mCherry allele to mark nuclei in a second strain. We first assessed the linear growth rate of strains in monoculture (Supplementary Fig. 3). For spore pairing experiments, spore counts from individual strains were normalized to 1.95 × 10^7^ conidia/mL and subsequently co-inoculated in a 1:1 ratio onto 30-cm race tubes in triplicate. Conidia at the beginning of the race tube were harvested on day 7 (“start”), which represented ∼1–2 days of growth, and at the “end” of the race tube on day 7, which represented 7 days of growth.

We first assessed pairings of strains that were of identical mating type, but where nuclei were differentially marked (hH1-GFP *mat A* or hH1-mCherry *mat A*); both these strains had identical linear growth rates (92.4 mm/day) (Supplementary Fig. 3). The inoculum for h1-GFP *mat A* and H1-mCherry *mat A* spores was normalized on day 0 in a near 1:1 ratio by flow cytometry (Fig. 1b), and conidial suspensions were inoculated in race tubes at the “inoculation site” (blue circle) of a 30-cm race tube (Fig. 1a). After 7 days, conidial suspensions at the “start” and “end” of the race tube were analyzed by flow cytometry (at least 10,000 spores). Fluorescence in the spores from the race tube was either green (presence of only GFP fluorescence), magenta (presence of only mCherry fluorescence), or both magenta and green (presence of both GFP and mCherry fluorescence) (Supplementary Fig. 2). From the h1-GFP *mat A* and H1-mCherry *mat A* conidial pairings, we observed a significant number of homokaryotic conidia, containing only H1-GFP or H1-mCherry signal, as well as ∼34% of heterokaryotic conidia containing both H1-GFP and H1-mCherry in conidial suspensions at both the “start” and “end” of the race tubes (Fig. 1b and c and Supplementary Fig. 2).

We then paired prototrophic conidia with conidia bearing an auxotrophic mutation; we postulated that on non-supplemented media, the population of auxotrophic nuclei would be outcompeted by wild-type nuclei in the syncytia and that nuclei containing the specific H1-tag associated with the auxotrophic mutation would be underrepresented in asexual spores. We also predicted that when the required exogenous supplementation was provided, the auxotrophic nuclei would increase in frequency in the spore suspensions compared to prototrophic nuclei. For these experiments, we used two auxotrophic mutants, one blocked in the ability to synthesize the vitamin B_5_ (pantothenic acid; *pan-2*) and a second mutant unable to synthesize the amino acid arginine (*arg-5*); both mutants were unable to grow on non-supplemented media. The linear growth rate of the *pan-2* mutant on supplemented media (93 mm/day) was not significantly different from WT (92.4 mm/day), but the *arg-5* mutant on supplemented media showed a moderately reduced linear growth rate (79.4 mm/day) (Supplementary Fig. 3).

To examine how auxotrophic genotypes behave in a heterokaryon, spores of the *Δpan*-2 or *Δarg-5* mutants were mixed in a 1:1 ratio with prototrophic spores and inoculated onto race tubes with or without supplementation (arginine + pantothenic acid) (Fig. 1a and b). Flow cytometry analysis of spore suspensions at the “start” and “end” of the race tubes on non-supplemented media showed that *Δpan-2* and *Δarg-5* nuclei were maintained throughout the colony as shown by the production of heterokaryotic spores, and homokaryotic *Δpan-2* or *Δarg-5* spores (Fig. 1c and d). However, in these pairings, prototrophic spores (Fig. 1c; H1-mCherry and Fig. 1d; H1-GFP) were significantly overrepresented in spore suspensions at both “start” and “end” of the race tube, particularly in comparison to *arg-5* spores. Homokaryotic auxotrophic spores were nevertheless regularly observed (∼22–29%) as well as heterokaryotic spore populations (∼26–35%) (Fig. 1b and c). These data indicated that nuclei unable to support colony growth due to their auxotrophic mutations, and thus dependent on the presence of WT nuclei for production of essential nutrients for survival, could still be compartmentalized in both homokaryotic and heterokaryotic spores.

We predicted that in pairings between *Δpan-2* conidia and *Δarg-5* conidia, that *Δpan-2* nuclei would be overrepresented in spore suspension at the end of the race tube due to the lower requirement for pantothenic acid for growth as compared to arginine, and that this result would be abolished with supplementation. To test this prediction, equal ratios of *Δpan-2* and *Δarg-5* conidia were inoculated into race tubes, and spore suspensions were evaluated by flow cytometry at the beginning and end of the race tubes. Homokaryotic *Δpan-2* spores showed a higher frequency as compared to *Δarg-5* spores at the start of the race tube experiments on non-supplemented media, but this discrepancy was abolished by the time syncytia grew to the “end” of race tubes (Fig. 1e). Under supplementation experiments in the prototrophic + *Δpan-2* pairings, the percentages of homokaryotic prototrophic and *Δpan-2* conidia were similar (∼25–28%) with a significant shift to heterokaryotic spores (∼45–48%) (Fig. 1f). Also, as predicted, under supplementation conditions, we observed a significant increase in the frequency of homokaryotic *Δarg-5* spores in prototrophic + *Δarg-5* pairings (Fig. 1g). To determine if this phenotype was particular to the *arg-5* mutation or was more broadly applicable to strains containing mutations in the arginine biosynthesis pathway, a second experiment was conducted by pairing prototrophic conidia with *Δarg-12* conidia (also an arginine auxotroph) (Supplementary Fig. 4). Similar to results with prototrophic + *Δarg-5* pairings on non-supplemented media (Fig. 1d), in prototrophic + *Δarg-12* pairings on non-supplemented media, ∼55% of spores were prototrophic, while *Δarg-12* nuclei were present in ≤10% of the homokaryotic spore population; ∼40% of the spores were heterokaryotic (Supplementary Fig. 4b). When arginine was added to the media, heterokaryotic spores increased in frequency to ∼62% in prototrophic + *Δarg-12* pairings; differences in the percentages of prototrophic vs *Δarg-12* homokaryotic conidia was abolished (Supplementary Fig. 4c). Thus, the arginine supplementation shift toward *Δarg-5* homokaryotic spores in prototrophic + *Δarg-5* pairings was not observed in prototrophic + *Δarg-12* pairings, although the overall effect of increasing the abundance of auxotrophic nuclei in asexual spores upon supplementation remained consistent. These data indicate that the nuclear ratios within a syncytium can be modified by external nutritional supplementation and mutational spectrum. Overall, these results showed that *N. crassa* syncytia are highly permissive toward harboring nuclei that “cheat” for nutrient supplementation and allowed the production of auxotrophic homokaryotic conidia despite these conidia being unable to germinate and grow on the media from which they were derived.

### Permissiveness for nuclear distribution mutants in syncytia

**Fig. 2.**
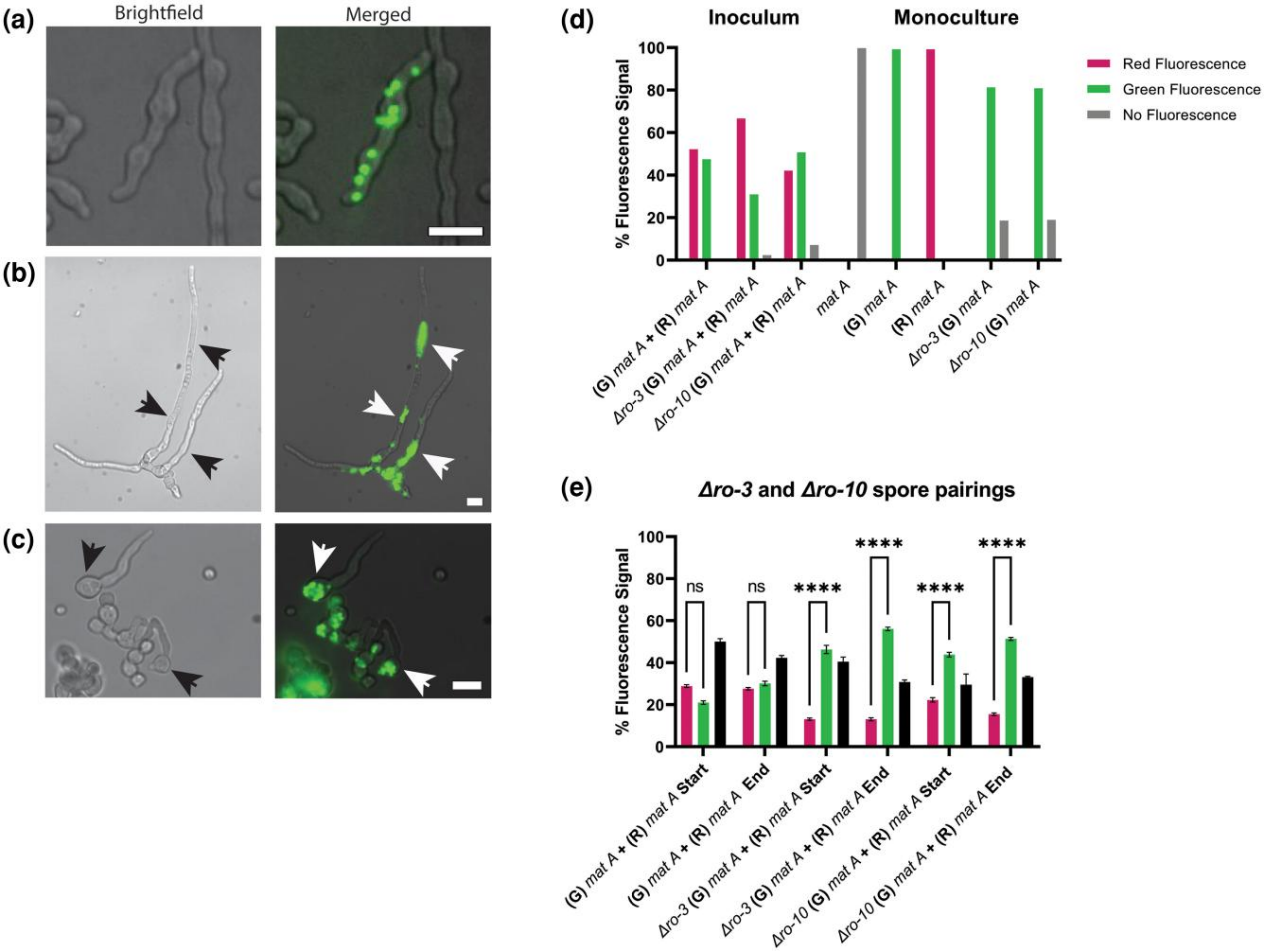
Flow cytometry analysis of spore pairings with nuclear spacing mutants. a) Normal nuclear spacing and morphology of the *his-3::hH1-eGFP mat A* (10BI) germlings. Adjacent cells in panel a without GFP fluorescence are *his-3::hH1-mCherry mat A* (74DM) germlings. b) Nuclear spacing defects of *Δro-3*, *his-3::hH1-eGFP mat A* (71AO) germlings. c) Nuclear spacing defects of *Δro-10*, *his-hH1-eGFP mat A* (72AO) germlings grown from spores at 3°C for approximately 6 hpi in liquid VMM on coverslips. Scale bar = 10 μm. d) Fluorescence signals (%) of each inoculum sample for every spore pairings used in this flow cytometry experiment (left side), and fluorescence signals (%) of monocultures from each strain used in this experiment, as well as an untagged *mat A* strain (FGSC 2489) (right side). e) *his-3::hH1-eGFP* (G) *mat A + his-3::hH1-mCherry* (R) *mat A* (10BI + 74DM), *Δro-3* (G) *mat A +* (R) *mat A* (71AO + 74DM), and *Δro-10* (G) *mat A* + (R) *mat A* (72AO + 74DM) spore pairings were grown in race tubes for 7 dpi at 3°C. Flow cytometry was conducted to analyze the relative percentage of each fluorescently tagged nuclear genotype in asexual spore populations derived from syncytia. Spore pairings in each biological replicate were derived from a single inoculum sample, and the ratios of each partner in the inoculum are shown in the “Inoculum” graph (panel d). “Start” denotes that spores were collected from the opening of the race tube most proximal to the inoculation site on day 7, and “End” denotes that the spore sample was collected from the portion of the race tube most distal from the site of the inoculation on day 7. Left (magenta) bars in graphs denote fluorescence signal from homokaryotic spores with the presence of only histone H1-mCherry-tagged nuclei, right (panel d) or middle (panel e) (green) bars in graphs denote fluorescence signal from homokaryotic spores with the presence of only histone H1-eGFP-tagged nuclei, black bars in graphs denote spores with signal from both histone H1-mCherry and histone H1-GFP nuclear tags in the same cell, and gray bars denote spores with no detectable fluorescence signal. Black and white arrows highlight the abnormal nuclear spacing in *ropy* mutants (panels b and c). Statistical analysis was conducted using two-way ANOVA followed by Tukey’s HSD post hoc tests. *P < 0.05; **P < 0.01; ***P < 0.001; ****P < 0.0001; ns = not significant. *N* = 4. Error bars = SEM. Pairwise post hoc t-tests between heterokaryotic spore populations of hH1-eGFP (G) *mat A*, *+* hH1-mCherry (R) *mat A* (10BI + 74DM) from the “END” and *Δro-3* (G) *mat A +* (R) *mat A* (71AO + 74DM) from the “END” were significantly different (*P* = 0.0003); Pairwise post hoc t-tests between heterokaryotic spore populations of hH1-eGFP (G) *mat A*, *+* hH1-mCherry (R) *mat A* (10BI + 74DM) from the “END”, and *Δro-10* (G) *mat A* + (R) *mat A* (72AO + 74DM) from “END” were significantly different (*P* = 0.0135).

Previous studies elucidated how bulk flow (or cytoplasmic streaming) contributes significantly to nuclear mixing within heterokaryotic hyphal compartments in *N. crassa* (Roper *et al.* 2013). In addition to bulk flow, dynein–dynactin motors also contribute to nuclear transport (Ramos-Garcia *et al.* 2009). Based on shared nutritional resources in syncytia shown by experiments in the previous section (Fig. 1), we predicted that if one nucleus were to be defective in motor-driven nuclear transport, when paired with WT nuclei, there would be little or no decrease in the fitness of either nucleotype. To test this hypothesis, we used two different (*ropy*) mutants, *Δro-3* and *Δro-10*; the former encodes for a subunit of dynein/dynactin motor protein complex, while the latter is responsible for localization/integrity of cytoplasmic dynein/dynactin (Bruno *et al.* 1996; Minke, Lee, and Plamann 1999; Minke, Lee, Tinsley *et al.* 1999; Xiang and Plamann 2003). Both mutants showed a severe nuclear spacing defect phenotype (Fig. 2b and c and Supplementary Fig. 5); bulk flow was previously shown to be sufficient to maintain some level of retrograde and anterograde nuclear movement (Ramos-Garcia *et al.* 2009). Consistent with their nuclear spacing defects, both the *Δro-3* and *Δro-10* mutants grew significantly slower than WT (28.5 mm/day and 26.9 mm/day, respectively, as compared to 92.4 mm/day for WT) (Supplementary Fig. 3). The *ro-3* and *ro-1* mutants showed a terminal linear growth phenotype on the race tubes at 8.1 and 8.6 cm of growth, respectively. However, WT + *Δro-3* and WT + *Δro-10* heterokaryons showed restored growth, as compared to the *Δro-3* and *Δro-10* strains in monoculture.

It was previously shown that *ropy* mutants exhibit ∼90% anucleate hyphal tips and ∼50% anucleate hyphal compartments (Plamann *et al.* 1994). Consistent with these findings, no nuclear fluorescence was observed in ∼15% of homokaryotic spores from the *Δro-3* or *Δro-10* mutants in monoculture (Fig. 2d); by comparison, homokaryotic WT strains displayed a small fraction (≤0.5%) of spores with no fluorescence. In pairings between *Δro-3* or *Δro-10* mutants and WT, the initial inoculum ratios evaluated by flow cytometry showed that ∼5–8% of spores lacked nuclear fluorescence (Fig. 2d). In contrast, conidial suspensions from the end of the race tube of the WT + *Δro-3* or WT + *Δro-10* pairings showed a low percentage of spores with no fluorescence (1.1 and 0.35%, respectively). The percentage of heterokaryotic spores in the WT + *Δro-3* and WT + *Δro-10* pairings was ∼30–35% (Fig. 2e). There was a significant increase in both *ro-3* and *ro-10* homokaryotic spores at both the “start” and “end” of the race tube relative to the initial inoculum levels (Fig. 2e). These data indicate that the *Neurospora* syncytia is highly permissive toward sharing of intracellular public goods, such as molecular motors, and that even nuclei with severe nuclear spacing defects have access to asexual spore formation.

### Mating type incompatibility leads to strong competitive selection and a “winner-takes-all” phenotype

It has been previously shown that opposite mating type strains (*mat A* and *mat a*) are required for mating and meiosis during the sexual cycle, but somatic cell fusion between *mat a + mat A* hyphae leads to HI, cell death, and breakdown of heterokaryotic colonies (Gross 1952; Metzenberg and Glass 1990; Glass and Kuldau 1992). Thus, we predicted that asexual spore pairings between *mat A* + *mat a* strains would result in a drastic reduction in heterokaryotic spores, and result in *mat A* or *mat a* homokaryotic spores in ratios representative of the initial inoculum.

**Fig. 3.**
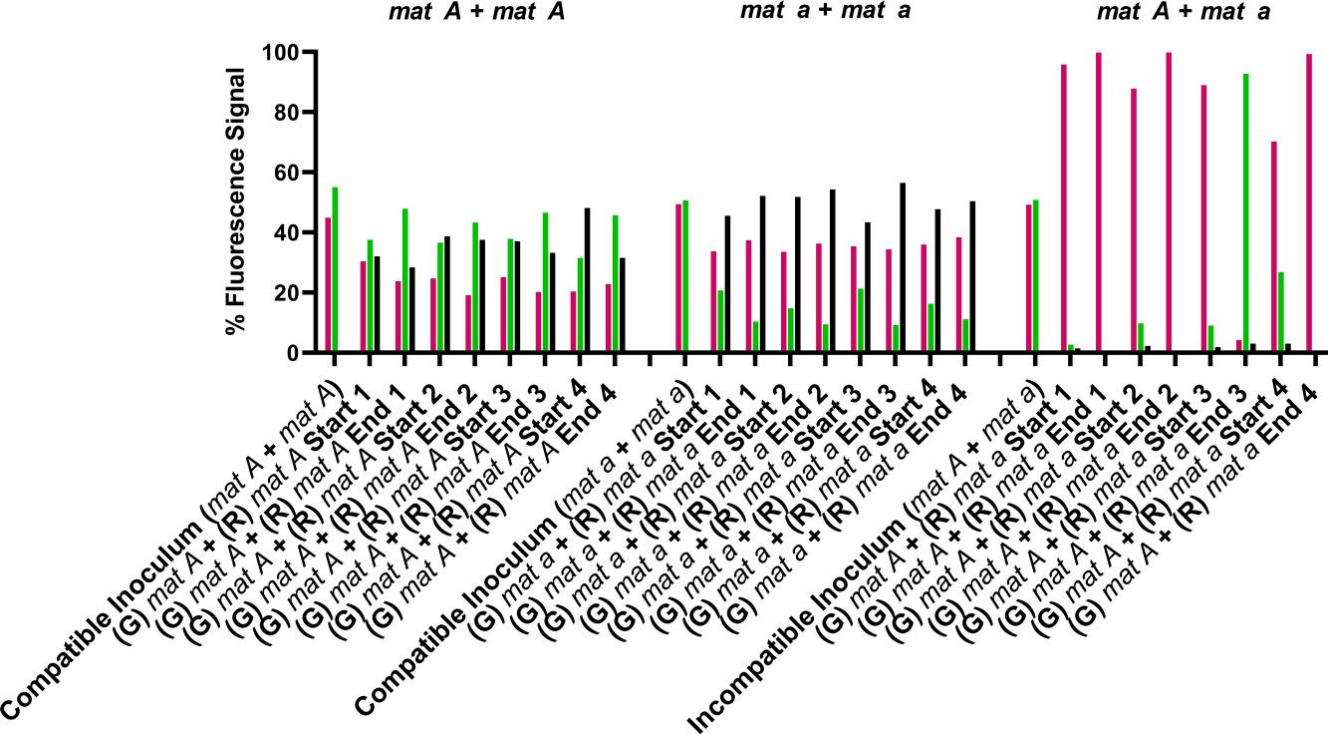
Individual flow cytometry runs of identical or opposite mating type spore pairings. Mating type compatible *his-3::hH1-eGFP* (G) *mat A + his-3::hH1-mCherry* (R) *mat A* spore pairings (10BI + 74DM) (left side), mating type compatible (G) *mat a +* (R) *mat a* spore pairings (8BH + 74ED) (middle), or mating type incompatible (G) *mat A +* (R) *mat a* (10BI + 74ED) spore pairings (right side) were inoculated in approximately equal ratios and grown in race tubes for 7 dpi at 3°C. Flow cytometry was conducted to analyze the relative percentage of each fluorescently tagged nuclear genotype in asexual spore populations derived from syncytia. All four biological replicates for each spore pairing are shown individually to clearly present which genotype nearly reached saturation in each race tube trial of the experiment. Spore pairings in each biological replicate were derived from a single inoculum sample, and the ratios of each partner in the inoculum are shown in the left-hand “Compatible Inoculum” and “Incompatible Inoculum” columns of each dataset in the graph. “Start” denotes that spores were collected from the opening of the race tube most proximal to the inoculation site on day 7, and “End” denotes that the spore sample was collected from the portion of the race tube most distal from the site of the inoculation on day 7. Left (magenta) bars in graphs denote fluorescence signal from homokaryotic spores with the presence of only histone H1-mCherry-tagged nuclei, right (inoculum) or middle (green) bars in graphs denote fluorescence signal from homokaryotic spores with the presence of only histone H1-eGFP-tagged nuclei, and black bars in graphs denote spores with signal from both histone H1-mCherry and histone H1-GFP nuclear tags in the same cell.

Individual compatible *mat A* + *mat A* or *mat a* + *mat a* spore pairings from single race tubes showed 28–56% heterokaryotic spores at the “start” and “end” of race tubes, as well as homokaryotic spores (Fig. 3). In contrast, *mat A* + *mat a* spore pairings showed a very small fraction (≤3%) of heterokaryotic spores, and a near “saturation” (≥92%) of one mating type nucleotype in homokaryotic spores (Fig. 3). The dominance, or near saturation of a particular *mat A* or *mat a* genotype in spore pairings, was termed as a “winner-takes-all” phenotype. Importantly, the nucleotype that showed near saturation in replicates was independent of mating type (although the *mat a* genotype “won” more often than the *mat A* genotype). Thus, contrary to our hypothesis, these results show that mating type incompatibility creates a clear selective advantage for one nuclear genotype in pairings of opposite mating type asexual spores.

**Fig. 4.**
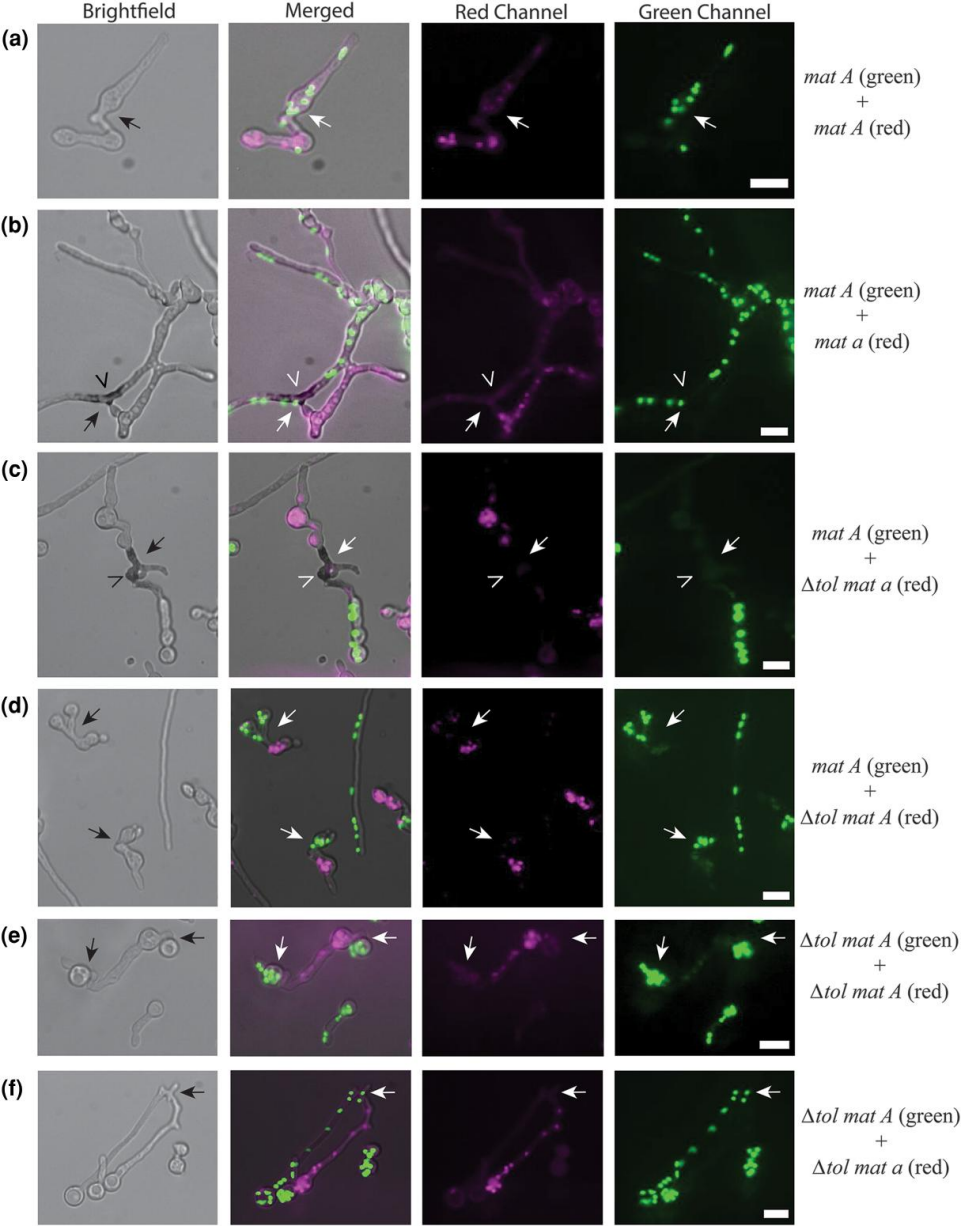
Vital dye staining of mating type compatible vs incompatible *Δtol* germlings. a) Mating type compatible *his-3::hH1-eGFP* (G) *mat A + his-3::hH1-mCherry* (R) *mat A* germlings (10BI + 74DM); b) mating type incompatible (G) *mat A* + (R) *mat a* germlings (10BI + 74ED); c) mating type incompatible (G) *mat A* + *Δtol* (R) *mat a* germlings (10BI + 76BI); d) mating type compatible (G) *mat A* + *Δtol* (R) *mat A* germlings (10BI + 76BM); e) mating type compatible *Δtol* (G) *mat A* + *Δtol* (R) *mat A* germlings (63FB + 76BM); and (F) mating type incompatible *Δtol* (G) *mat A* + *Δtol* (R) *mat a* germlings (63FB + 76BI) grown from spores at 3°C for approximately 6 hpi, followed by staining with methylene blue vital dye (Brightfield Channel), and observed by fluorescence microscopy. Representative images from at least 3 independent experiments are shown. Red fluorescence channel false-colored magenta and overlay of green + magenta may appear whiter in “Merged” images. Black and white arrows denote regions between germlings where anastomosis had occurred, and open arrow heads highlight hyphal compartments showing positive vital dye staining. Scale bar = 10 μm.

Although mating type incompatibility has been investigated in colonies, it has not previously been evaluated in germlings undergoing somatic cell fusion. To visualize germling fusion events occurring at the “start” of the race tubes, we conducted vital dye staining using MB and fluorescence microscopy of mating type compatible (*mat A* + *mat A*) vs incompatible (*mat A* + *mat a*) spore pairings (Fig. 4a and b). We observed evidence of mating type incompatibility via vacuolization and dark blue MB staining of fused hyphal compartments between opposite mating type germlings, starting at ∼6 hpi (Fig. 4b). Mating type compatible germlings (*mat A* + *mat A*) showed no evidence of MB staining post-fusion (Fig. 4a).

### TOL, required for mating type incompatibility, is not nuclear-limited and suppresses the “winner-takes-all” phenotype

Previous findings showed that loss-of-function mutations at the *tol* (for “tolerant”) locus are sufficient to suppress mating type incompatibility in heterokaryons (Newmeyer 1970); *tol* mutations have no effect on sexual reproduction (Griffiths 1982; Saupe *et al.* 1996; Shiu and Glass 1999). The *tol* locus encodes a protein containing a HET domain, which is of unknown biochemical function, but is a motif commonly observed in proteins associated with HI (Paoletti and Clave 2007). We first assessed the phenotype of fused germlings by fluorescence microscopy on *Δtol* mutant spore pairings to determine if *mat A Δtol* + *mat a Δtol* pairings showed any evidence of death, as observed with *mat A* + *mat a* pairings (Fig. 4b). Pairings of germlings with identical mating type (*mat A + Δtol mat A* and *Δtol mat A* + *Δtol mat A*) showed no evidence of cell death following fusion, as expected (Fig. 4d and e). Pairings between *Δtol mat A* + *Δtol mat a* germlings also did not show any evidence of cell death, consistent with the ability of mutations at *tol* to suppress mating type incompatibility (Fig. 4f). However, vital dye staining of *Δtol mat a* + *mat A* spore pairings revealed that fused cells showed vacuolization and death (dark blue staining pattern in “Brightfield Channel’) starting at approximately 6–8 hpi (Fig. 4c), with a phenotype similar to fusion between *mat A* + *mat a* germlings (Fig. 4b). These data are consistent with previous results showing that mutations in *tol* are recessive (Newmeyer 1970; Jacobson 1992).

**Fig. 5.**
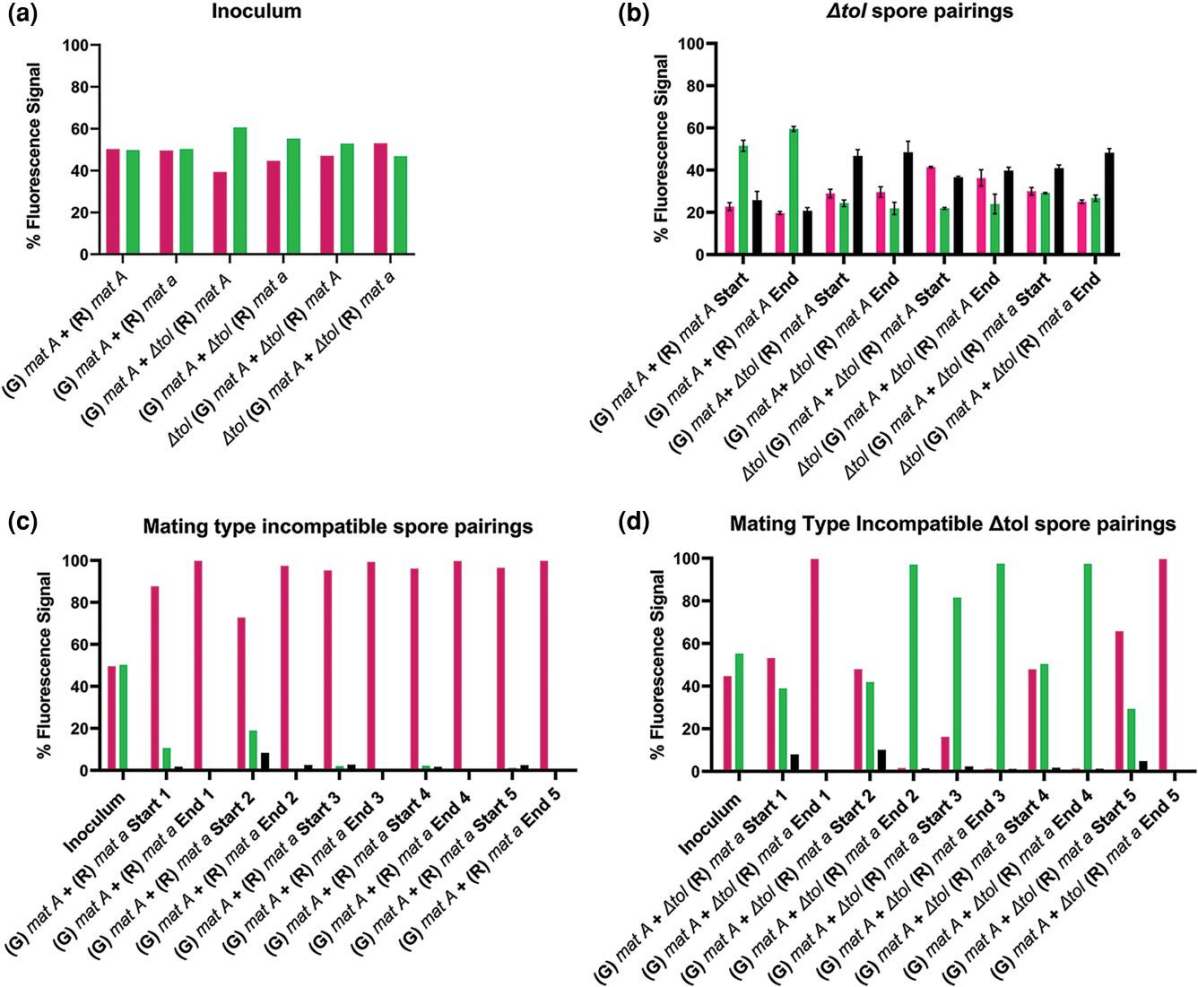
Flow cytometry analysis of spore pairings with mating type compatible and incompatible *Δtol* strains. Spores were inoculated into race tubes in approximately equal ratios and grown for 7 dpi at 3°C. a) Inoculum ratios for all spore pairings used in this flow cytometry experiment. b) Identical mating type *his-3::hH1-GFP* (G) *mat A* + *his-3::hH1-mCherry* (R) *mat A* (10BI + 74DM) spore pairings; identical mating type (G) *mat A* + *Δtol* (R) *mat A* (10BI + 76BM) spore pairings; identical mating type *Δtol* (G) *mat A* + *tol* (R) *mat A* (63FB + 76BM) spore pairings; and opposite mating type *Δtol* (G) *mat A* + *Δtol* (R) *mat a* (63FB + 76BI) spore pairings. c) Individual biological replicates from opposite mating type spore pairings of (G) *mat A* + (R) *mat A* (10BI + 74DM). d) (G) *mat A* + *Δtol* (R) *mat a* (10BI + 76BI). Flow cytometry was conducted to analyze the relative percentage of each fluorescently tagged nuclear genotype in asexual spore populations derived from syncytia. “Start” denotes that spores were collected from the opening of the race tube most proximal to the inoculation site on day 7, and “End” denotes that the spore sample was collected from the portion of the race tube most distal from the site of the inoculation on day 7. Left (magenta) bars in graphs denote fluorescence signal from homokaryotic spores with the presence of only histone H1-mCherry-tagged nuclei, right (inoculum) or middle (green) bars in graphs denote fluorescence signal from homokaryotic spores with the presence of only histone H1-eGFP-tagged nuclei, and black bars in graphs denote spores with signal from both histone H1-mCherry and histone H1-GFP nuclear tags in the same cell. All five biological replicates for each spore pairing are shown individually in panels c and d to clearly present which genotype nearly reached saturation in each race tube trial of the experiment. Statistical analysis was conducted using two-way ANOVA followed by Tukey’s HSD post hoc tests. *P < 0.05; **P < 0.01; ***P < 0.001; ****P < 0.0001; ns = not significant. *N* = 5. Error bars = SEM.

We predicted that if both partners in a mating type incompatible heterokaryon also carried a *Δtol* mutation, spore pairings of identical mating type (*Δtol mat A* + *Δtol mat A*) or opposite mating type (*Δtol mat A* + *Δtol mat a*) would show similar frequencies of H1-GFP, H1-mCherry, and heterokaryotic spores as assessed by flow cytometry. Consistent with this prediction, *mat A + mat A*, *Δtol mat A* + *mat A*, *Δtol mat A* + *Δtol mat A*, and *Δtol mat A* + *Δtol mat a* spore pairings did not display a winner-takes-all phenotype. For the *Δtol mat A* + *Δtol mat a* spore pairing in particular, both nucleotypes were found in homokaryotic spores and also showed a significant fraction (38–54%) of heterokaryotic spores (Fig. 5b). These results show that the *tolerant* mutation was sufficient to suppress the “winner-takes-all” phenotype observed in opposite mating type pairings and that the incompatibility function of mating type was essential for this phenotype. In contrast, all pairings between spores of opposite mating type, but where the *tol* mutation was in only one partner, showed a similar winner-takes-all phenotype as opposite mating type spore pairings (Fig. 5c and d). Importantly, in some cases, the wild-type genotype won, while in others, the *tol* mutant genotype won, showing that there was no bias for nuclear origin of *tol*. These data support our finding that mating type incompatibility leads to partitioning of nuclear genotypes into individual homokaryons by failure to maintain viable heterokaryotic syncytia. Furthermore, these results support the hypothesis that mating type incompatibility mediated by TOL produces a barrier to nuclear mixing of opposite mating types, which not only serves the function of allorecognition but consequently produces a distinct competitive advantage for one nucleotype.

### The winner-takes-all phenotype is not limited to mating type incompatibility

A major aspect of HI mediated by allelic differences at *het* loci is cell permeabilization and death of the fusion compartment after somatic cell fusion (Saupe 2000; Glass and Kaneko 2003). Our data indicates that mating type incompatibility results in a winner-takes-all phenotype when opposite mating type spores are co-inoculated. We hypothesized that the induction of cell death drives this “winner-takes-all” phenotype. We therefore assessed whether genetic differences at a different *het* locus, the *regulator of cell death-1* (*rcd-1*) locus (Daskalov *et al.* 2019; Daskalov *et al.* 2020), also result in a winner-takes-all outcome in incompatible pairings.

The *rcd-1* locus encodes two allelic variants, *rcd-1-1* and *rcd-1-2*. When *rcd-1-1* and *rcd-1-2* germlings undergo anastomosis, they rapidly (∼20 minutes post-fusion) exhibit vacuolization, permeabilization, and cell death (Daskalov *et al.* 2019); mating and sexual development are unaffected in crosses between strains that are incompatible for *rcd-1*. Microscopy of germling fusion cells showed clear vacuolization in both mating type compatible and mating type incompatible *rcd-1-1* + *rcd-1-2* paired germlings (≤1 hour) 6 hpi post-fusion, as previously reported (Supplementary Fig. 6). RCD-1 encodes a functional homolog of mammalian gasdermin, which regulates a cell death process termed pyroptosis (Daskalov *et al.* 2020; Daskalov and Glass 2022).

**Fig. 6.**
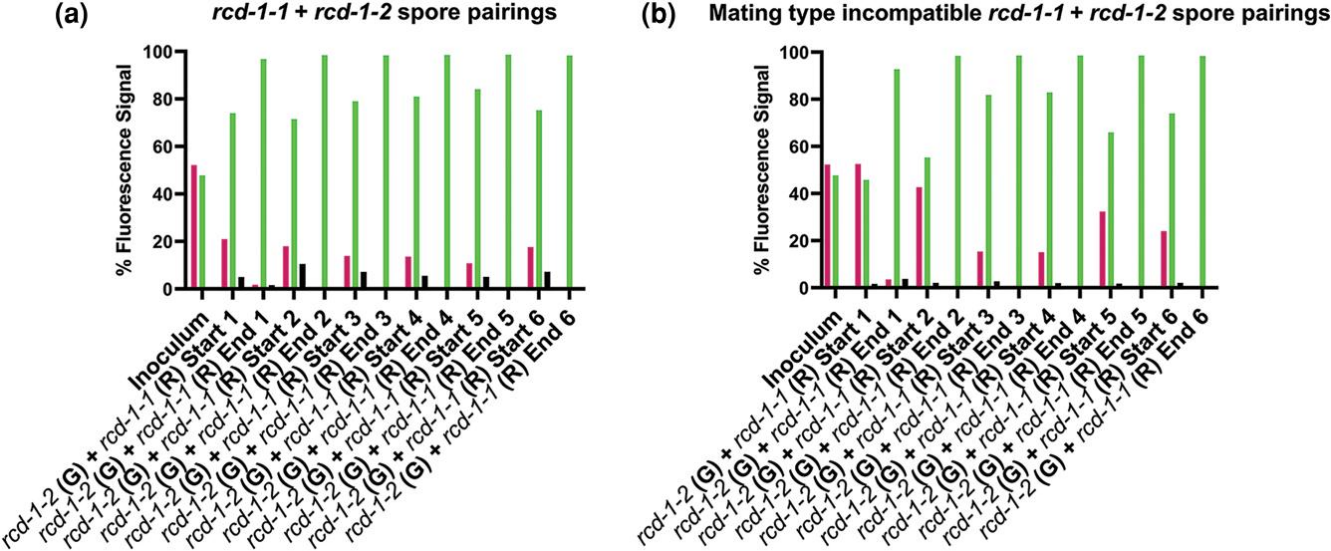
Flow cytometry analysis of spore pairings between *rcd-1-1 + rcd-1-2* of identical and opposite mating type. a) Individual biological replicates of mating type compatible *csr-1::rcd-1-2 his-3::eGFP Δrcd-1* (G) *mat A + rcd-1-1 his-3::hH1-mCherry* (R) *mat A* (5AB + 74DM) spore pairings. b) Individual biological replicates of mating type incompatible *csr-1::rcd-1-2* (G) *Δrcd-1 mat A + rcd-1-1* (R) *mat a* (5AB + 74ED) spore pairings. Spores were inoculated in approximately equal ratios and grown in race tubes for 7 dpi at 3°C. Flow cytometry was conducted to analyze the relative percentage of each fluorescently tagged nuclear genotype in asexual spore populations. Spore pairings in each biological replicate were derived from a single inoculum sample, and the ratios of each partner in the inoculum are shown in the first “Inoculum” column of each graph. “Start” denotes that spores were collected from the opening of the race tube most proximal to the inoculation site on day 7, and “End” denotes that the spore sample was collected from the portion of the race tube most distal from the site of the inoculation on day 7. Left (magenta) bars in graphs denote fluorescence signal from homokaryotic spores with the presence of only histone H1-mCherry-tagged nuclei, right (inoculum) or middle (green) bars in graphs denote fluorescence signal from homokaryotic spores with the presence of only histone H1-eGFP-tagged nuclei, and black bars in graphs denote spores with signal from both histone H1-mCherry and histone H1-GFP nuclear tags in the same cell. All six biological replicates for each spore pairing are shown individually to clearly present which genotype nearly reached saturation in each race tube trial of the experiment.

If regulated cell death is the major driver of the nuclear dominance phenotype, we predicted that pairings between incompatible *rcd-1* strains would also exhibit a winner-takes-all phenotype. To test this hypothesis, we paired equal ratios of otherwise isogenic wild-type *rcd-1-1* strain with a *Δrcd-1* strain with the *rcd-1-2* allele targeted to the *csr-1* locus and of identical mating type onto race tubes and evaluated the frequency of each genotype in asexual spores at the start and the end of the race tube. Pairings between *rcd-1-1* + *rcd-1-2* strains showed a very similar winner-takes-all phenotype as mating type incompatible spore pairings (Fig. 3), with a clear dominance of one nuclear genotype (*rcd-1-2*) (Fig. 6a). Spore pairings between strains that were of opposite mating type and different at *rcd-1* (*rcd-1-1 hH1-mCherry mat a + rcd-1-2*; cytoplasmic-GFP *mat A*) also showed a winner-takes-all phenotype that was not significantly different than pairings between *rcd-1 + rcd-1-2* of identical mating type (Fig. 6b). These data show that regulated cell death (RCD) mediated by allelic differences at *het* loci such as mating type or *rcd-1* is a major driver that enables one genotype to outcompete another in spore pairings between incompatible strains.

### A block in hyphal fusion partially recapitulates the “winner-takes-all” phenotype

RCD triggered by allelic differences at *het* loci during somatic growth in filamentous fungi first requires cell fusion (Saupe 2000; Fischer and Glass 2019). We predicted that a block in cell fusion, which would eliminate cell death, would abolish the “winner-takes-all” phenotype of mating type incompatible and *rcd-1* incompatible pairings. To test this hypothesis, we used two strategies. The first strategy used strains with allelic differences at *cwr* (*cell wall remodeling*), a locus that encodes two linked genes (*cwr-1* and *cwr-2*) that regulate cell wall dissolution and germling/hyphal fusion in *N. crassa* (Gonçalves et al. 2019; Detomasi *et al.* 2022); post-fusion phenotypes (such as cell death) are not regulated by genetic differences at the *cwr locus*. *cwr-1* alleles from one of the six CWR haplogroups found in *N. crassa* populations result in a block in somatic cell fusion when paired with germlings bearing *cwr-2* from any of the other CWR haplogroups. For example, paired germlings expressing incompatible *cwr-1/cwr-2* alleles from different haplogroups (for example, *cwr-1^HG1^* + *cwr-2^HG6^*) show a block in cell wall dissolution during somatic cell fusion events (Gonçalves *et al.* 2019; Detomasi *et al.* 2022). In a second strategy, we used a mutant termed *soft (so)*, which has a drastic defect in chemotropic interactions and somatic cell fusion with itself and with wild-type cells (Fleißner *et al.* 2005).

For the *cwr-1/cwr-2* experiments, we used the haplogroup 1 genotype (*cwr-1*^HG1^  *cwr-2*^HG1^) paired with an otherwise isogenic strain, but that contained an incompatible *cwr-1* allele from a haplogroup 2 strain (*his-3::cwr-1*^HG2^  *Δcwr-2*), as well as an otherwise isogenic strain containing an incompatible *cwr-1* allele from a haplogroup 6 strain (*his-3::cwr-1*^HG6^  *Δcwr-2*). As a control, we also paired the *cwr-1*^HG1^  *cwr-2*^HG1^ strain with a *his-3:: cwr-1^HG1^ Δcwr-2* strain. The strain carrying *cwr-2*^HG1^ (*cwr-1*^HG1^  *cwr-2*^HG1^) causes a cell fusion block when interacting with cells carrying either *cwr-1*^HG2^ or *cwr-1*^HG6^ (Gonçalves *et al.* 2019; Detomasi *et al.* 2022). Germlings with compatible *cwr-1/cwr-2* alleles (*cwr-1*^HG1^  *cwr-2*^HG1^ + *his-3:: cwr-1^HG1^ Δcwr-2*) showed robust fusion (∼90% of interacting germlings), while *cwr-1^HG1^ cwr-2^HG1^* germlings paired with either *cwr-1^HG2^ Δcwr-2* or *cwr-1^HG6^ Δcwr-2* germlings showed cell fusion frequencies of ∼14% and ∼5% of interacting germlings, respectively (Gonçalves *et al.* 2019; Detomasi *et al.* 2022). Thus, we predicted that in incompatible *cwr* pairings, the block in cell fusion would suppress the “winner-takes-all” phenotype of pairings between incompatible cells due the reduction in fusion and thus cell death with a concomitant reduction in heterokaryotic spores. Additionally, we predicted that homokaryotic spore genotypes in spore suspensions from the race tubes would reflect the initial proportions of inoculated spore genotypes.

**Fig. 7.**
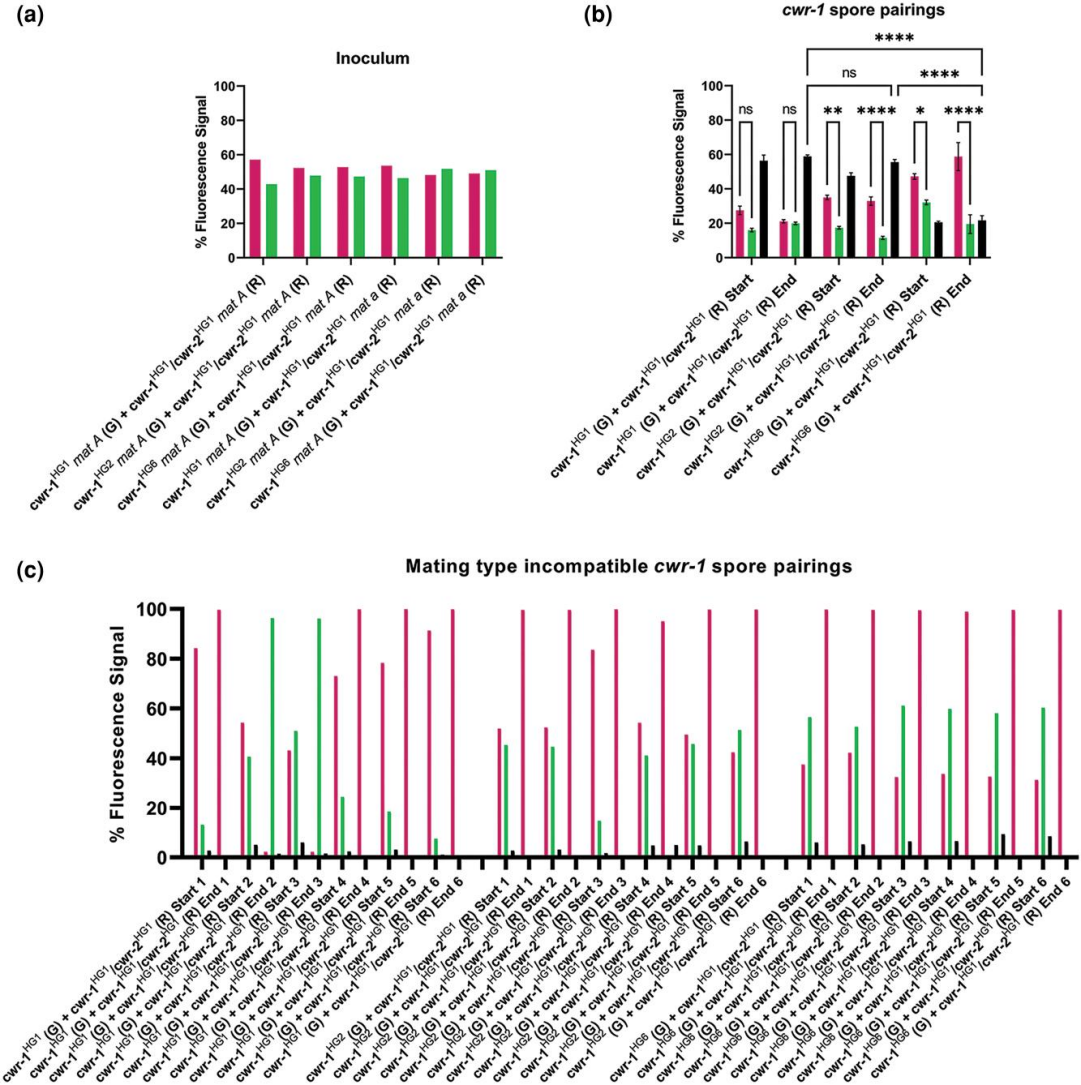
Flow cytometry analysis of spore pairings with mating type compatible and incompatible *cwr-1^HG1^*, *cwr-1^HG2^*, and *cwr-1^HG6^* in pairings with a *cwr-1^HG1^ cwr-2^HG2^* strain. (a) Inoculum ratios for all spore pairings used in this flow cytometry experiment. b) Mating type compatible spore pairings *cwr-1^HG1^ cwr-2^HG1^ his-3::hH1-mCherry* (R) *mat A* (74DM) with *cwr-1^HG1^ Δcwr-2 his-3::*eGFP (G) *mat A* (336a) or *cwr-1^HG2^ Δcwr-2* (G) *mat A* (88a) or *cwr-1^HG6^ Δcwr-2* (G) *mat A* (385a) strains. c) Individual replicates of the mating type incompatible spore pairings of *cwr-1^HG1^ cwr-2^HG1^*(R) *mat a* (74ED) + *cwr-1^HG1^ Δcwr-2* (G) *mat A* (336a) or *cwr-1^HG2^ Δcwr-2* (G) *mat A* (88a), or *cwr-1^HG6^ Δcwr-2* (G) *mat A* (385a). Each strain was inoculated in approximately equal ratios and grown in race tubes for 7 dpi at 3°C. Flow cytometry was conducted to analyze the relative percentage of each fluorescently tagged nuclear genotype in asexual spore populations derived from syncytia. Spore pairings in each biological replicate were derived from a single inoculum sample. “Start” denotes that spores were collected from the opening of the race tube most proximal to the inoculation site on day 7, and “End” denotes that the spore sample was collected from the portion of the race tube most distal from the site of the inoculation on day 7. Left (magenta) bars in graphs denote fluorescence signal from homokaryotic spores with the presence of only histone H1-mCherry-tagged nuclei, right (panel a) or middle (panels b and c) (green) bars in graphs denote fluorescence signal from homokaryotic spores with the presence of only histone H1-eGFP-tagged nuclei, and black bars in graphs denote spores with signal from both histone H1-mCherry and histone H1-GFP nuclear tags in the same cell. All six biological replicates for each mating type incompatible spore pairing are shown individually to clearly present which genotype nearly reached saturation in each race tube trial of the experiment. CWR haplogroup 1 (HG1); haplogroup 2 (HG2); and haplogroup 6 (HG6) naming from Gonçalves *et al.* (2019) and Detomasi *et al.* (2022). Statistical analysis was conducted using two-way ANOVA followed by Tukey’s HSD post hoc tests. *P < 0.05; **P < 0.01; ***P < 0.001; ****P < 0.0001; ns = not significant. *N* = 6. Error bars = SEM.

Spore pairings between *cwr-1*^HG1^  *cwr-2*^HG1^ + *cwr-1*^HG2^  *Δcwr-2* showed no significant differences in the percentages of homokaryotic or heterokaryotic spores from fully compatible *cwr* spore pairings (*cwr-1*^HG1^  *cwr-2*^HG1^ + *cwr-1*^HG1^  *Δcwr-2*) (Fig. 7b). However, *cwr-1*^HG1^  *cwr-2*^HG1^ + *cwr-1*^HG6^  *Δcwr-2* spore pairings, which have a more severe reduction in cell fusion (∼95%) (Detomasi *et al.* 2022), showed a significant reduction in the percentage of heterokaryotic spores in spore suspensions from both the start and end of the race tubes (Fig. 7b). From these data, we predicted that pairings between *cwr-1*^HG1^  *cwr-2*^HG1^  *mat A* + *cwr-1*^HG6^  *Δcwr-2 mat a* strains would not exhibit the drastic winner-takes-all phenotype associated with mating type incompatibility (Fig. 3), due the reduction in cell fusion, cell death, and production of heterokaryotic spores. However, in spore pairings of opposite mating type, the *cwr-1^HG1^ cwr-2^HG1^* nucleotype was dominant when paired with spores of *cwr-1^HG2^ Δcwr-2* or *cwr-1^HG6^ Δcwr-2* genotypes (Fig. 7c), although a significant percentage of homokaryotic *cwr-1^HG6^ Δcwr-2* spores was present in spore suspensions at the start of the race tube. These data show that the significant reduction in cell fusion observed in incompatible CWR pairings was not sufficient to abolish the “winner-takes-all” phenotype of mating type incompatible pairings and suggests that the post-fusion cell death function of *het* loci is an important driver for this phenotype.

**Fig. 8.**
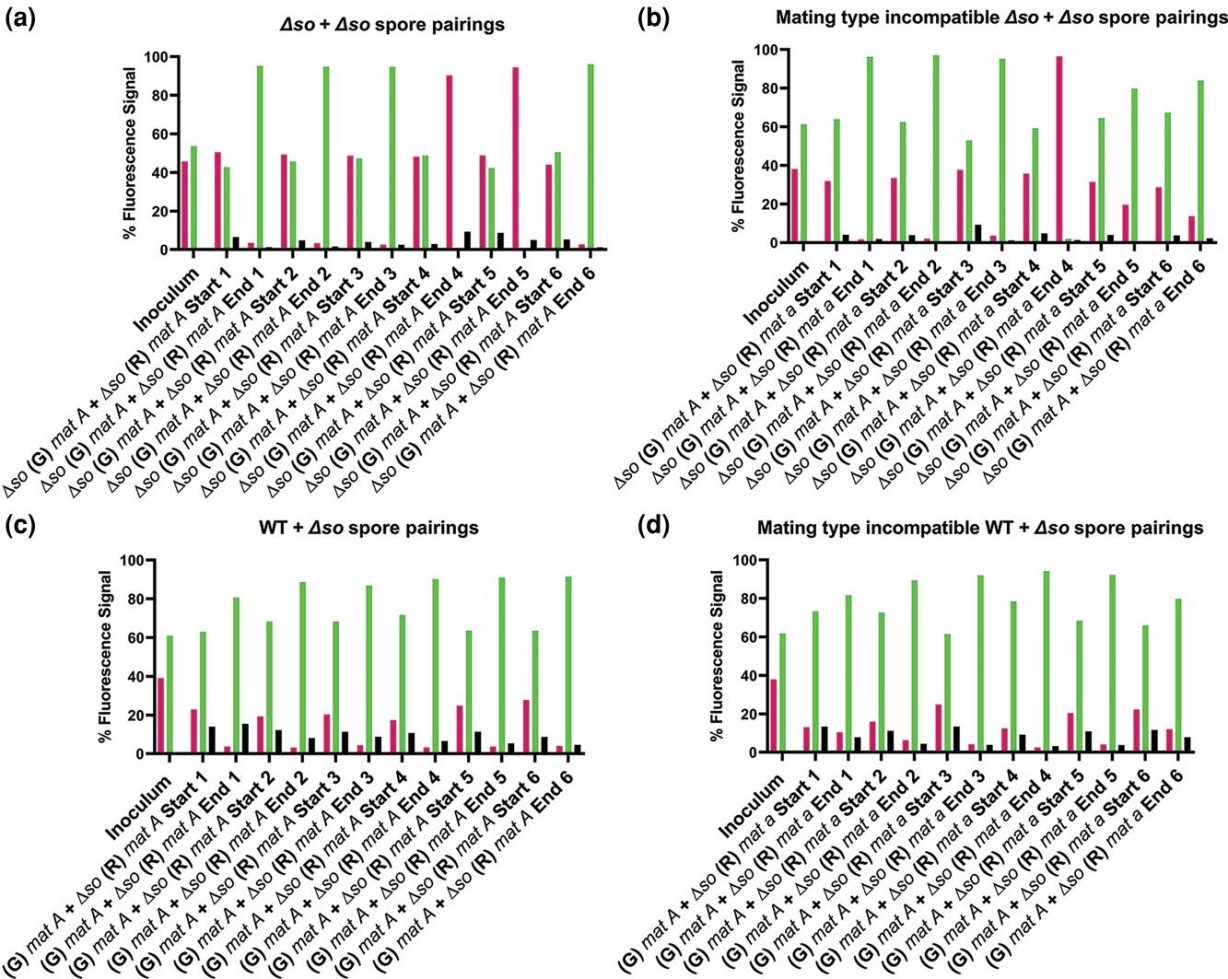
Flow cytometry analysis of mating type compatible and incompatible *Δso + Δso* spore pairings. a) Individual biological replicates of mating type compatible spore pairings with *Δso his-3::hH1-eGFP* (G) *mat A* + *Δso his-3::hH1-mCherry* (R) *mat A* (19C + 80AS). b) Mating type incompatible spore pairings with *Δso* (G) *mat A* + *Δso* (R) *mat a* (19C + 80BB). c) Mating type compatible spore pairings with *Δso* (R) *mat A* + (G) *mat A* (80AS + 10BI). d) Mating type incompatible spore pairings of *Δso* (R) *mat a* + (G) *mat A* (80BB + 10BI). Conidia from these strains were inoculated in approximately equal ratios in race tubes and grown for 7 dpi at 3°C. Flow cytometry was conducted to analyze the relative percentage of each fluorescently tagged nuclear genotype in asexual spore populations. Spore pairings in each biological replicate were derived from a single inoculum sample, and the ratios of each partner in the inoculum are shown in the first “Inoculum” column of each graph. “Start” denotes that spores were collected from the opening of the race tube most proximal to the inoculation site on day 7, and “End” denotes that the spore sample was collected from the portion of the race tube most distal from the site of the inoculation on day 7. Left (magenta) bars in graphs denote fluorescence signal from homokaryotic spores with the presence of only histone H1-mCherry-tagged nuclei, right (inoculum) or middle (green) bars in graphs denote fluorescence signal from homokaryotic spores with the presence of only histone H1-eGFP-tagged nuclei, and black bars in graphs denote spores with signal from both histone H1-mCherry and histone H1-GFP nuclear tags in the same cell. *N* = 6.

The incompatible *cwr* strains showed a significant reduction, but not a complete block in cell fusion, and did not suppress the winner-takes-all phenotype of opposite mating type strains. We therefore chose to also assess interactions using a strain that is blocked in cell fusion, *soft (so)*. Null mutants in *so* are unable to undergo chemotrophic interactions resulting in ∼300-fold reduction in somatic cell fusion (Fleissner *et al.* 2005). The SOFT protein oscillates at the tips of cells undergoing chemotropic interactions (Fleissner *et al.* 2009; Fleissner and Herzog 2016) and has been shown to be a scaffold for the upstream components of the Cell Wall Integrity MAPK signaling pathway (Teichert *et al.* 2014; Weichert *et al.* 2016). We reasoned that there should be an extremely low proportion of heterokaryotic spores produced in *Δso* + *Δso* pairings; moreover, without the cell death associated with post-fusion HI, we predicted that homokaryotic nuclear populations from race tube spore suspensions would reflect initial spore genotype frequencies in the inoculation samples. Flow cytometry of *Δso mat A (R)* + *Δso mat A (G)* spore pairings of identical mating type showed nearly equal ratios of both spore genotypes at the start of the race tube, but, unexpectedly, showed only one nucleotype at the end of the race tube (Fig. 8a). These results were similar to mating type incompatible *Δso mat a (R)* + *Δso mat A (G)* pairings (Fig. 8b). However, in replicates 5 and 6 of the *Δso mat a* + *Δso mat A* pairings, neither nuclear genotype reached saturation, albeit the nucleotype in the slight majority in the inoculum was more represented [≥75%; *Δso mat A (G)*] in the final spore suspension. As predicted, a very small proportion (≤5%) of the spore population was heterokaryotic in the *Δso mat A (R)* + *Δso mat A (G)* pairings and the opposite mating type *Δso mat a (R)* + *Δso mat A (G)* pairings.

Previous data showed that *Δso* and nuclei containing mutations in other loci required for somatic cell fusion mutants are present in evolved strains that “cheat” their way into spores (Grum-Grzhimaylo *et al.* 2021). The *Δso* mutant showed a minor growth defect (76.4 mm/day) in linear growth as compared to wild-type cells (92.4 mm/day) (Supplementary Fig. 3). We hypothesized that the wild-type germlings/colonies would be able to fuse with themselves, and potentially with adjacent *Δso* germlings/colonies, while *Δso* germlings/colonies would be unable to fuse with themselves or wild-type cells, resulting in a selective advantage for wild type as compared to *Δso* nucleotypes. Consistent with this prediction, in both mating type compatible and incompatible WT + *Δso* spore pairings, we observed that the WT genotype showed a “winner-takes-all” phenotype (Fig. 8c and d). Post hoc t-tests showed that the percentage of heterokaryotic spores in either mating type compatible or incompatible (WT + *Δso*) or (*Δso* + *Δso*) spore pairings, or with mating type incompatible (*mat A* + *mat a*) spore pairings, was not statistically different. The WT + *Δso* pairings showed a clear bias for the WT genotype at the “start” of the race tubes, whereas in experiments with *Δso* + *Δso* pairings, the spore ratios at the “start” of the race tubes were very similar to the initial starting inoculum. These data show that the winner-takes-all phenotype can be driven not only by post-fusion cell death but can also occur when cells of different genotypes are unable to undergo somatic cell fusion.

## Discussion

Genetic cooperation and conflict on an organismal level have been an important topic of study in biology, starting most notably with Hamilton's studies of social insects (Hamilton 1964). More recently, investigations on microbes have revealed mechanisms of cooperation and conflict (West *et al.* 2007). Organelles, such as nuclei, mitochondria, and chloroplasts, may act as semi-autonomous units within an organism, encoding distinct genetic information and thus providing additional levels of genomic conflict within cells and syncytia, beyond inter-organismal social structures (Dobrogojski *et al.* 2020; Kouvelis and Hausner 2022). Here, we show that the network of *N. crassa* is permissive to the generation and maintenance of defective nuclei within a syncytium, as long as WT nuclei were present. WT nuclei essentially “cooperate” with defective nuclei by providing resources that the defective nucleus cannot produce. Syncytia facilitate dispersion of these nuclei and WT intracellular public goods primarily via bulk flow, and are independent of nuclear genotype, resulting in the production of both homokaryotic and heterokaryotic spores. This capacity is a type of “bet hedging”, such that beneficial mutations within the syncytium can be carried to the next generation either as homokaryotic spores or as heterokaryotic spores, and where mutant nuclei could potentially be subjected to further evolution via mutational events within the resulting heterokaryotic syncytia. Bet hedging has been previously used to explain heterogeneity in asexual spore size and germination rates in *Aspergillus fumigatus* based on fluctuations in environmental conditions from which the spores were derived (Kang *et al.* 2021). This aspect has also been explored in artificial evolution experiments in *N. crassa*, by systematically analyzing sexual spore germination and viability under alternating time periods of favorable and unfavorable environmental conditions (Graham *et al.* 2014).

In contrast to the highly permissive nature of sharing intracellular goods in spore pairings of auxotrophic and *ropy* mutants with WT, here we show that permissiveness is limited to syncytia that have genetic identity at post-fusion *het* loci. Allorecognition mediated by genetic differences at *het* loci and which result in cell death following somatic cell fusion has been shown to be important for the reduction in transfer of mycoviruses and defective mitochondria between genetically different strains in a number of filamentous fungal species (Debets *et al.* 1994; Liu and Milgroom 1996; van Diepeningen *et al.* 1997; Debets and Griffiths 1998; Zhang *et al.* 2014; Wu *et al.* 2017), and which has been postulated to be a type of fungal innate immunity (Paoletti and Saupe 2009; Daskalov and Glass 2022; Gaspar and Pawlowska 2022). In *N. crassa*, there are at least 14 allorecognition loci that result in cell death following somatic cell fusion (Zhao *et al.* 2015), thus potentially generating >2 million incompatible genotypes in segregating populations (Goncalves and Glass 2020). Here, we show that RCD mediated by genetic differences at the *mat* and *rcd-1* loci not only causes death of fusion compartments but results in a winner-takes-all phenotype in pairings of incompatible strains. A study assessing fitness effects in *N. crassa* showed a similar trend of competitive exclusion in pairings between opposite mating type strains (Kronholm *et al.* 2020). In addition, we show that the cell-death inducing trigger mediates this competitive advantage, as mutations at a locus (*tol*) required for mating-type mediated cell death abolished the winner-takes-all phenotype. In mating type incompatible pairings, the *mat a* strain often won (Figs. 3c, 5c and 7c). It is unclear what the basis of the *mat a* strain's capacity to triumph over the *mat A* strain in these pairings; a similar dominance was also observed for the *rcd-1-2* strain in pairings with *rcd-1-1* (*rcd-1-2 mat A)*. It is possible that slight differences in germination rate, germling/hyphal fusion frequencies, or the strength of the death response in compartments surrounding the fusion cell could play a role. Interestingly, the bias of *mat a* over *mat A* was not observed in the *Δso mat A + Δso mat a* pairings (Fig. 8). It would be of interest to determine if the bias of *mat a* strains over *mat A* strains (and *rcd-1-2* over *rcd-1-1*) in competitive pairings also occurs under conditions more akin to those found in nature. Nevertheless, in population samples, the frequencies of *mat A*, *mat a*, *rcd-1-1*, and *rcd-1-2* alleles are nearly equal (Daskalov *et al.* 2019).

To further test whether fusion-mediated cell death or a block in cell fusion was essential for the winner-takes-all phenotype, we tested pairings between strains that were incompatible at the *cwr-1* and *cwr-2* loci, which significantly reduces somatic cell fusion frequencies, but where cell death is not triggered post-fusion; these pairings showed significant proportions of heterokaryotic spores (Fig. 7). These data indicate that HI resulting in cell death has a here-to-fore underappreciated role in competition for resources between genetically incompatible strains and which may also be involved in contributing to the evolution of allelic diversity observed in these systems (Saupe 2000; Muirhead *et al.* 2002; Goncalves and Glass 2020).

The characterized *het* loci in filamentous fungi do not affect sexual fertility; indeed, *N. crassa* is an obligate outbreeder and crosses with strains that are genetically different at many *het* loci. Post-fusion incompatible interactions in outbreeding species mediated by genetic differences at *het* loci must somehow be suppressed during sexual reproduction, as cell fusion and proliferation of opposite mating type nuclei in a common cytoplasm are a prerequisite for karyogamy and subsequent meiosis (Raju 1980; Berteaux-Lecellier *et al.* 1998). HI mediated by opposite mating types during vegetative growth is not unique to *Neurospora* species, but has also been reported in distantly related species, such as *Ascobolus stercorarius* (Bistis 1994). In the pseudohomothallic species, *Podospora anserina*, in which ascospores are dikaryotic, genetic differences at *het* loci can result in meiotic drive and ascospore abortion (Dalstra *et al.* 2003; Debets *et al.* 2012) as well as reproductive isolation (Ament-Velásquez et al. 2022).

Previous studies have investigated the existence of “nucleus-limited” genes in several fungal model systems, where a nucleus containing the WT allele of a gene cannot complement a null mutant allele when paired in a heterokaryon (Newmeyer 1970; Demeter *et al.* 2000; Czaja *et al.* 2013; Kasbekar 2014). Here, we show that the *Δtol* mutation suppressed mating type incompatibility in heterokaryons only where both partners lacked a functioning copy of *tol*. If one partner was *tol+*, regardless of mating type, this was sufficient to complement the null mutation and induce mating type incompatibility, thereby recapitulating the “winner-takes-all” phenotype (Fig. 5). These data support the hypothesis that TOL is not a nucleus-limited protein.

The *soft* mutants, which are defective in chemotropic interactions and somatic cell fusion and are blocked in cell death in mating type incompatible spore pairings, also showed a similar dominance of one nuclear genotype in spore populations. The severely reduced proportion of heterokaryotic spores in mating type compatible and mating type incompatible *Δso* + *Δso* pairings suggests that competition occurred predominantly between homokaryotic *Δso* colonies. In WT + *Δso* spore pairings, the dominant nuclear genotype was consistently WT. The growth advantage of WT over *Δso* (Supplementary Fig. 3) and the ability to undergo anastomosis resulting in more rapid spore production are presumably the cause of WT consistently prevailing over *Δso* strains. In a number of filamentous fungal species, such as in the human pathogen *A. fumigatus*, somatic cell fusion is rare (Macdonald *et al.* 2019), thus, these strains would mimic *Δso* mutants in nature. We predict that pairings between fusion deficient *A. fumigatus* strains would also show a winner-takes-all phenotype that is likely independent of genetic differences at *het* loci. The interplay in the regulation of somatic cell fusion vs post-fusion allorecognition likely impacts life history evolution in filamentous ascomycete species.

Here, we show that *N. crassa* syncytia are highly permissive to replication and maintenance of mutant nuclei, allowing these nucleotypes to compete for access to asexual spores. One interesting finding was that *Δarg-*5 homokaryotic spores had a higher representation than WT in syncytia when arginine was provided, even though *Δarg-*5 strains with supplementation had a slightly lower growth rate than WT (Supplementary Fig. 3). However, when a strain carrying a different *arg* mutation (*Δarg-12)* was paired with WT on supplemented media, we did not see a similar increase in *Δarg-12* homokaryotic spores, rather an increase in mixed spores, reflective of an increase in the *Δarg-12* nucleotype. In *N. crassa*, arginine is largely sequestered to the vacuoles, which regulates the levels of cytosolic arginine (Weiss and Davis 1977; Davis 1986). Vacuolar distribution in syncytia follows similar heterogeneous patterns of localization as nuclei (Bowman *et al.* 2009, 2015), where cytoplasmic flow and microfluidic eddies result in uneven distribution of these organelles throughout the mycelial network (Pieuchot *et al.* 2015). Our data suggests that utilization of resources within heterokaryotic syncytia is complex. Localization of nuclei, as well as whether syncytial public goods are accessed cytoplasmically, within organelles (e.g. vacuoles, the endoplasmic reticulum, and mitochondria), or even extracellularly by leakage or secretion, may affect both competition and sharing of resources within a syncytia.

## Supplementary Material

iyad112_Supplementary_Data

## Data Availability

Strains developed for this study are available from the Fungal Genetics Stock Center (Supplementary Table 1). All data necessary for confirming the conclusions presented in the article are represented fully within the article. Raw flow cytometry data files are available upon request. Supplementary tables and figures are available at Figshare (https://doi.org/10.6084/m9.figshare.22908764).
