# Supplementary material for: Permissiveness and competition within and between Neurospora crassa syncytia
Source: Genetics. 2023 Jun 14;224(4):iyad112. doi: 10.1093/genetics/iyad112 (PMC10411585; doi:10.1093/genetics/iyad112)
Supplement: iyad112_Supplementary_Data [file iyad112_supplementary_data.pdf]

Supplemental Tables and Figures

Permissiveness and Competition within and between *Neurospora crassa* Syncytia

Alexander P. Mela[a] and N. Louise Glass[a,b]

[a] The Plant and Microbial Biology Department, The University of California, Berkeley, CA 94720, [b] The Environmental Genomics and Systems Biology Division, The Lawrence Berkeley National Laboratory, Berkeley, CA 94720

Table S1. Strains used in this study

Name	Genotype	Source
**2489**	*mat A*	FGSC 2489
**4200**	*mat a*	FGSC 4200
**6103**	*Δhis-3 mat A*	FGSC 6103
**10BI**	*his-3::hH1-eGFP mat A*	This Study<br>FGSC 27258
**93DR**	*his-3::hH1-eGFP mat A*	This Study
**8BH**	*his-3::hH1-eGFP mat a*	This Study<br>FGSC 27257
**74DM**	*his-3::hH1-mCherry mat A*	This Study<br>FGSC 27265
**74ED**	*his-3::hH1-mCherry mat a*	This Study<br>FGSC 27266
**11U**	*his-3::hH1-eGFP; Δpan-2 mat A*	This Study<br>FGSC 27259
**102CC**	*his-3::hH1-mCherry; Δarg-5 mat A*	This Study<br>FGSC 27271
**131AK**	*his-3::hH1-eGFP; Δarg-12 mat A*	This Study<br>FGSC 27272
**71AO**	*his-3::hH1-eGFP; Δro-3 mat A*	This Study<br>FGSC27263
**72AO**	*his-3::hH1-eGFP; Δro-10 mat A*	This Study<br>FGSC 27264
**76BI**	*his-3::hH1*-mCherry; *Δtol mat a*	This Study<br>FGSC 27267
**76BM**	*his-3::hH1*-mCherry; *Δtol mat A*	This Study<br>FGSC 27268
**63FB**	*his-3::hH1*-eGFP; *Δtol mat A*	This Study<br>FGSC 27262
**57CE**	*his-3::hH1*-eGFP; *Δtol mat a*	This Study<br>FGSC 27261
**Fluffy A7**	*Δfl mat a*	This Study
**Fluffy BF**	*Δfl mat A*	This Study
**336a**	*his-3::cwr-1^{2489}; Δcwr-1 ΔNCU01381 Δcwr-2::hyg^{R}, csr-1:: GFP, mat A*	Goncalves *et al.* 2019;<br>Detomasi *et al.* 2022
**88a**	*his-3::cwr-1^{D111}; Δcwr-1 ΔNCU01381 Δcwr-2::hyg^{R}, csr-1:: P-ccg-1-GFP mat A*	Detomasi *et al.* 2022<br>FGSC 27123
**385a**	*his-3::cwr-1^{JW228}; Δcwr-1 ΔNCU01381 Δcwr-2::hyg^{R}, csr-1:: Pccg-1-GFP mat A*	Goncalves *et al.* 2019
**5AB**	*his-3::Pccg-1-GFP csr-1::rcd-1-2; Δrcd-1-1 mat A*	This Study<br>FGSC27256
**19C**	*his-3::hH1-eGFP; Δso mat A*	This Study<br>FGSC 27260
**80AS**	*his-3::hH1-mCherry; Δso mat A*	This Study<br>FGSC 27269
**80BB**	*his-3::hH1-mCherry; Δso mat a*	This Study<br>FGSC 27270

Table S2. Primers used in this study

Primer Name	Sequence 5'-3'	Notes
hH1 Forward Primer 1	F-ATGCCTCCCAAGAAGACCGAGACCA	Includes hH1 Start codon; excludes Pccg1 Flank
hH1 Reverse Primer 2	R-CGAGCCCGCTGCCGAGAAGGCA	Excludes hH1 stop codon and glycine linker
5X Glycine Linker	GGAGGAGGAGGAGGA	Inserted between hH1 and mCherry sequences
mCherry Forward Primer 3	F-CAAGACACAGCCTGGGGTAATGG	Includes mCherry start codon in primer sequence
Tccg1 Reverse Primer 4	R-CGGCTAATGGGGTCTGAATGC	Includes Tccg1 Terminator
pmf272-Pccg1-hH1-PF11	F-CGTCACAGACAACGGGTGAAGG	sequencing Pccg1-hH1 fragment
pmf272-Pccg1-hH1-PF10	F-GCACTTCCATTGGGACAGGC	sequencing Pccg1-hH1 fragment
hH1-mCherry Sequence-PF8	F-GGCAACGTCACCGACAACATG	sequencing hH1-Linker-mCherry fragment
pmf272- insertion_Tccg1 PF1	F-CTAGGAATTGACCCGGCTCTCTG	sequencing mCherry-Tccg1 fragment
pmf272- insertion_Tccg1 PR7	R-GCGTGATGAACTTCGAGGACGG	sequencing hH1-Linker-mCherry fragment
pmf272-PF9 Insertion Check	F-TTGGCACCTCCTCCACTTCTCC	Check for hH1 insertion into pmf272
pmf272-PR10 Insertion Check	R-GCACCTCATCTGCTGGAATAGGG	Check for pmf272 into genome
pm272 Insertion Primer Forward 1	F-CTGAGAAGGAGGGCATCAAGCC	Verification for pmf272 insertion into genome
pmf272_Pccg1 Insertion PR5	R-AACAGAAAAGCACACAGACAGCG	Verification for pmf272 insertion into genome at his-3 flank

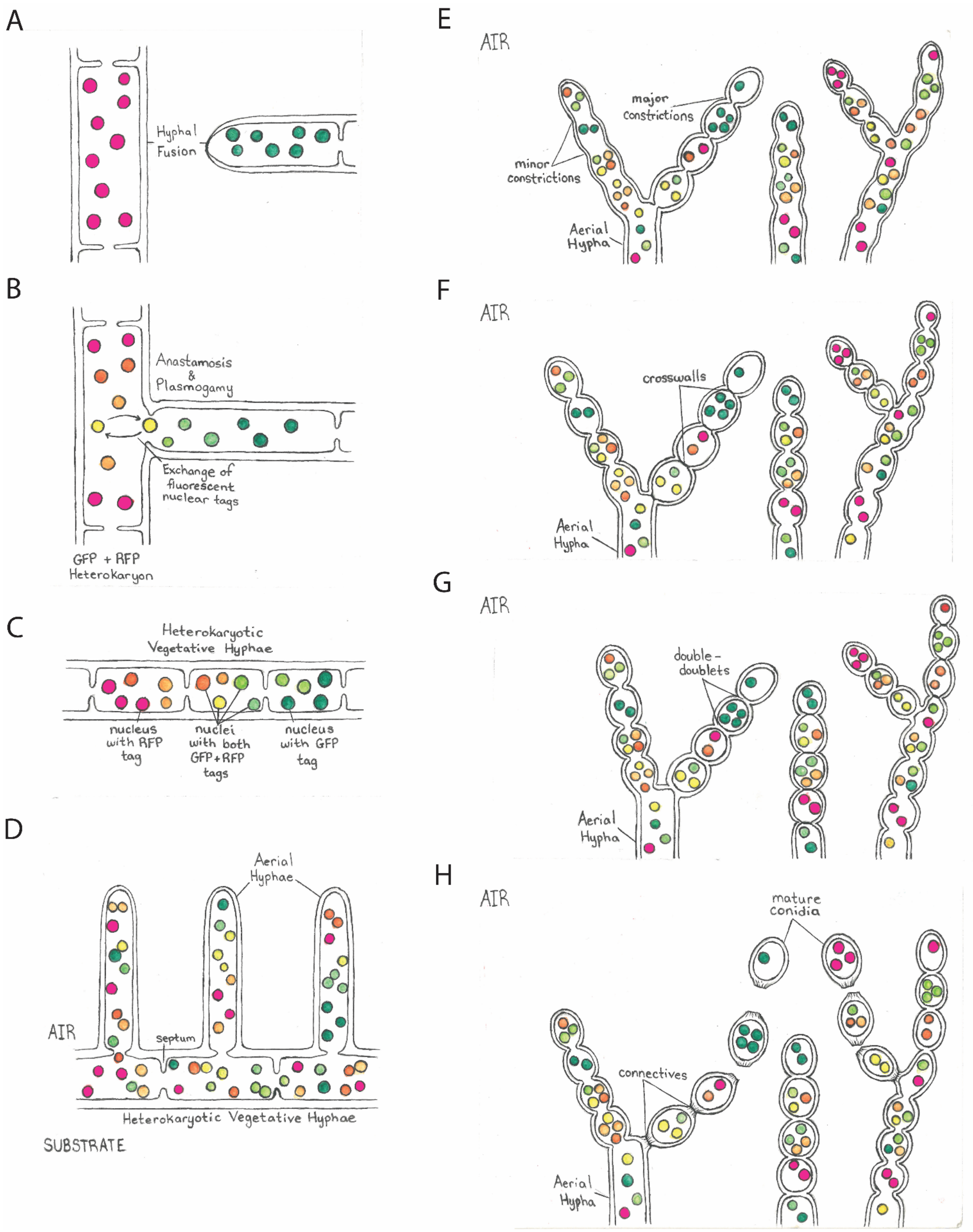

Figure S1. Sporogenesis and nuclear arrangement in *Neurospora crassa*. (A) Chemoattraction of vegetatively compatible homokaryotic hyphae containing differentially marked nuclei promotes growth towards one another, and subsequent (B) hyphal fusion, followed by exchange of fluorescent nuclear tags in a common cytoplasm. (C) Heterokaryotic vegetative hyphal compartments containing a heterogenous population of fluorescently labeled, genetically distinct nuclei, allow passage of free-flowing cytoplasm and organelles through septal pores; (D) vegetative hyphal compartments embedded in/on a 'substrate' give rise to the growth of aerial hyphae into an 'air' interface, that on average, have been recorded to reach heights of 6.2-7.1mm (+/- 1mm) above the growth medium (aerial hyphae reduced in length relative to other hyphal structures for best fit in Panels D-H) (Ma *et al.* 2016). Nuclei

from adjacent cells subsequently migrate throughout these growing aerial structures. (E) A hallmark of early stage conidiation is the presence of ‘minor constrictions’ of the aerial hyphae cell walls, which invaginate and demarcate future conidium cells (proconidia); in later stages of sporulation, elongation of cells budding from minor constriction chains give rise to more compartmentalized proconidium cells termed, ‘major constrictions’; (F) single ‘crosswalls’ of cell wall material form between developing spores, encapsulating cytoplasm and nuclei; followed by development of (G) mature double-layered cell walls, termed ‘double-doublets’. (H) Remaining cell wall material or, ‘connectives’, hold chains of developing spores together until mature conidia are released through gentle physical perturbations or other means. Green indicates H1-GFP nuclei; Magenta indicates H1-mCherry nuclei; yellow to orange indicate nuclei that contain both H1-GFP and H1-mCherry.

A

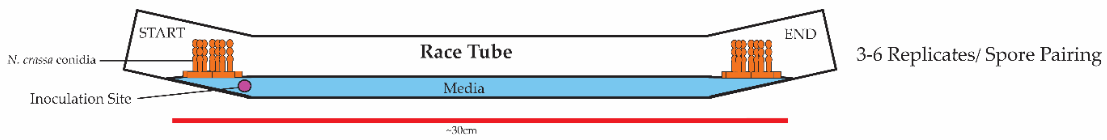

B

Analyzed on Day 0

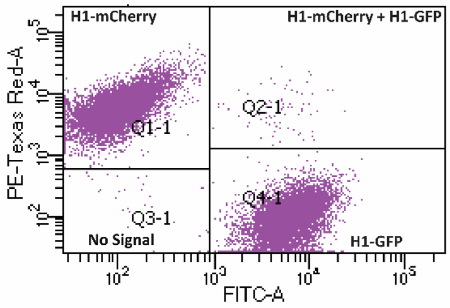

Tube: **hH1-GFP, *mat A* + hH1-mCherry, *mat A* spores from inoculum sample**

Population	#Events	%Parent	%Total
All Events	20,000	####	100.0
P1	18,630	93.2	93.2
P2	17,847	95.8	89.2
Q1-1	8,574	48.0	42.9
Q2-1	82	0.5	0.4
Q3-1	32	0.2	0.2
Q4-1	9,159	51.3	45.8

C

Analyzed on Day 7

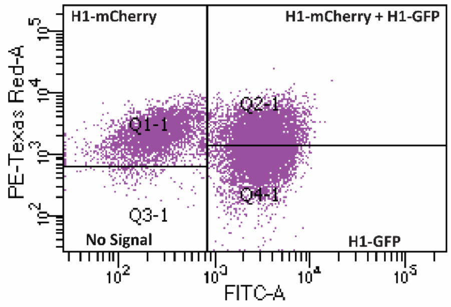

Tube: **hH1-GFP, *mat A* + hH1-mCherry, *mat A* spores from 'END'**

Population	#Events	%Parent	%Total
All Events	20,000	####	100.0
P1	16,083	80.4	80.4
P2	13,494	83.9	67.5
Q1-1	3,803	28.2	19.0
Q2-1	4,725	35.0	23.6
Q3-1	158	1.2	0.8
Q4-1	4,808	35.6	24.0

D

Analyzed on Day 7

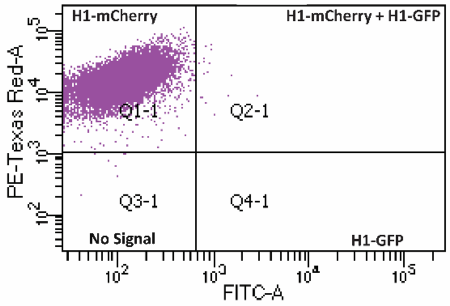

Tube: **hH1-GFP, *mat A* + hH1-mCherry, *mat a* spores from 'END**

Population	#Events	%Parent	%Total
All Events	20,000	####	100.0
P1	17,710	88.6	88.6
P2	15,422	87.1	77.1
Q1-1	15,396	99.8	77.0
Q2-1	17	0.1	0.1
Q3-1	9	0.1	0.0
Q4-1	0	0.0	0.0

E

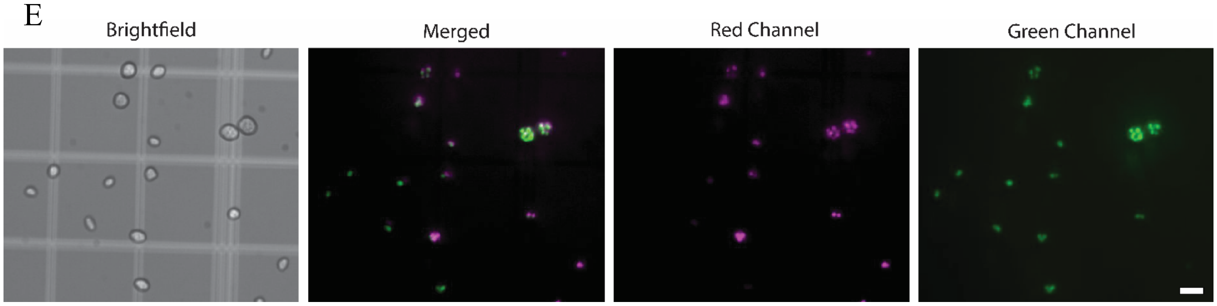

Figure S2. Schematic of race tube inoculation, harvesting, and flow cytometry analysis. (A) Diagram of a race tube, with 'START' and 'END' of the unit labeled. Sterile media (blue) with or without supplementation covers approximately 30cm of the bottom of the race tube interior. Spores (orange) develop throughout race tube, with the highest concentration of sporulation occurring at each end of the race tube. On Day 0, spores are normalized to the same spore concentration, combined in a 1:1 ratio (unless otherwise noted), analyzed by flow cytometry and subsequently inoculated at the 'inoculation site' (magenta circle) near the 'START' of the race tube. (B-D) After calibrating voltages with control samples of each individual strain used in the experiment, at least 10k events from the sample of interest were counted by flow cytometery (All Events) for each replicate sample per spore pairing. Cells are gated for spore size/granularity and doublets based on [side scatter area x forward scatter area] and [forward scatter height x forward scatter area] respectively (P1), to avoid counting spores too large/small/granular, cell debris, and cells stuck together (doublets). The data were further grouped in a two-parameter density plot, based on fluorescence signal in 4 quadrants (P2). The PE-Texas Red filter detects hH1-mCherry fluorescence & FITC filter detects hH1-GFP fluorescence. Purple dots on scatter plot (Left) represent the individual cells counted after gating and grouping. Q1-Red Fluorescence Signal (hH1-mCherry), Q2-Red + Green Fluorescence (hH1-mCherry + hH1-GFP), Q3- No Fluorescence (No detectable mCherry or GFP fluorescence signal), and Q4- Green Fluorescence (hH1-GFP). The read-out (Right) of the population hierarchy for the number of all events counted, or in other words how many spores/particles passed through the laser (#Events); a hierarchy of the percentage of the population of spores counted in each quadrant after gating and grouping for all parameters mentioned above (%Parent); and the percentage of the total events counted overall after gating and grouping for all parameters mentioned above (%Total). (B) Example read-out of the flow cytometry data from the inoculum sample of a (*his-3::hH1-eGFP mat A* (10BI) + *his-3::hH1-mCherry mat A* (74DM)) spore pairing analyzed on Day 0. (C) Example read-out of flow cytometry data shown here is a representative sample harvested on Day 7, and is from the 'END' of a race tube from (*his-3::ehH1-GFP mat A* (10BI) + *his-3::hH1-mCherry mat A* (74DM)) identical mating type spore pairing. (D) Example read-out of flow cytometry data shown here is a representative sample from Day 7, and is from the 'END' of a race tube containing a (*his-3::ehH1-GFP mat A* (10BI) + *his-3::hH1-mCherry mat a* (74ED)) opposite mating-type spore pairing. (E) Spores of a *his-3::hH1-eGFP* (G) *mat A* + *his-3::hH1-mCherry* (R) *mat A* (10BI +74DM) spore pairing harvested from the 'END' of a race tube, inoculated onto a hemocytometer, and imaged under 20X magnification for the presence of red (false-colored magenta) and green nuclear fluorescence. Merged channel shows spores with both red and green fluorescence (overlay may appear whiter). Representative image of conidia derived from a typical race tube experiment in this study. Scale Bar = 10 μm.

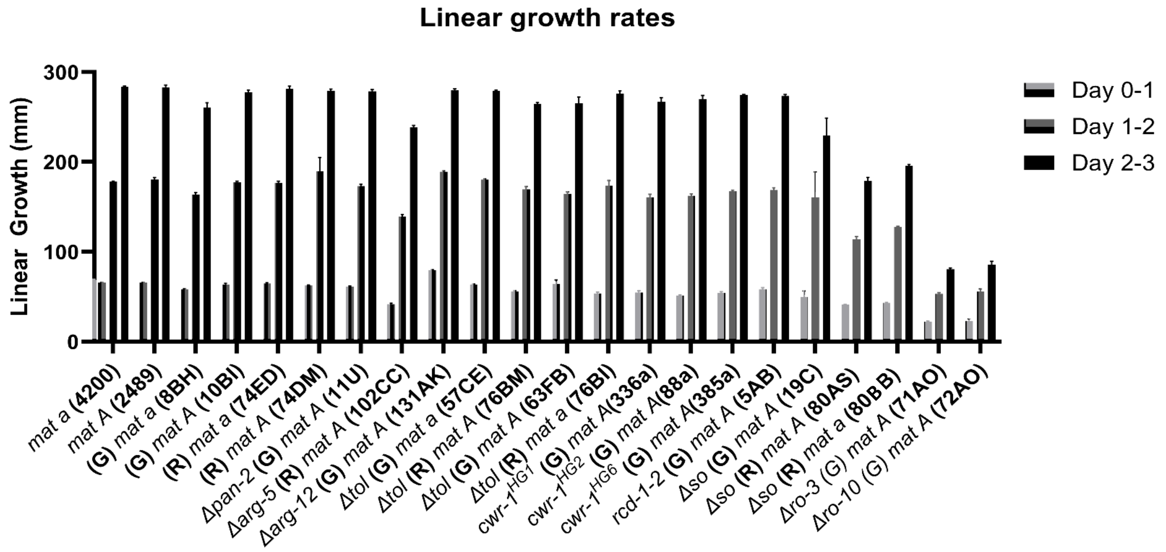

Figure S3. Linear growth rates of strains used in this study. Linear growth rates (mm) were measured for strains used in this study over three days in race tubes with the same media and growth conditions used in previous flow cytometry experiments. Strain names are shown in parenthesis. Histone H1-GFP or cytoplasmic-GFP tag (G); Histone H1-mCherry (R). Statistical analyses were conducted using two-way ANOVA followed by Tukeys HSD post hoc tests. Two-way posthoc t-tests between strain 4200 Day 2-3 and 8BH Day 2-3, p-not significant; 102CC Day 2-3-p<0.0001; 131AK Day 2-3-p>0.9999; 19C Day 2-3-p=0.9891; 80AS Day 2-3-p<0.0001; 80BB Day 2-3-p<0.0001; 71AO Day 2-3-p<0.0001; and 72AO Day 2-3-p<0.0001. N=3. Error Bars = SEM.

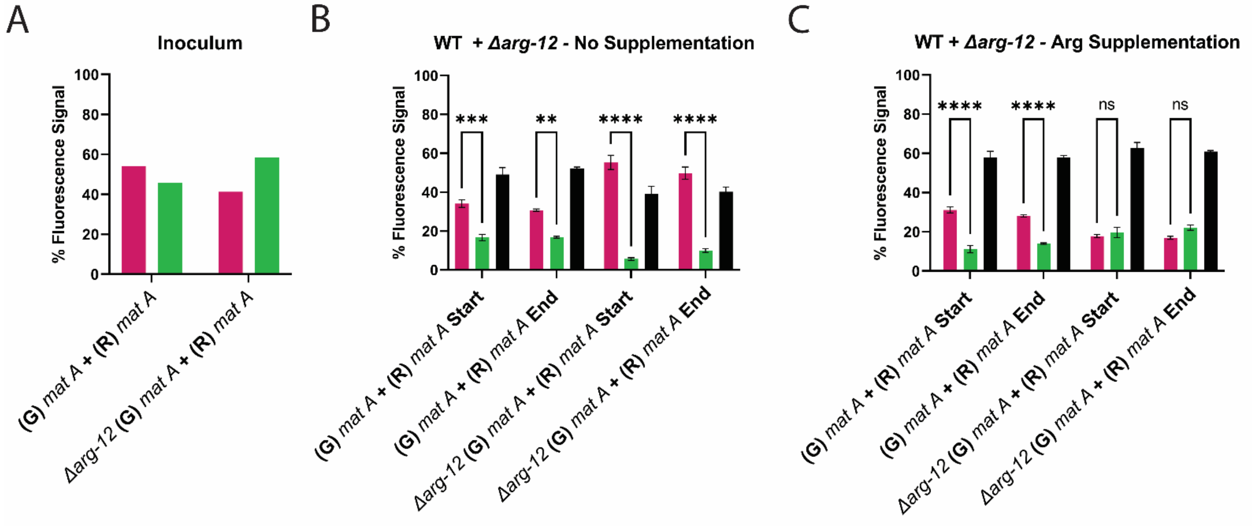

Figure S4. Flow cytometry analysis of prototrophic and auxotrophic spore pairings with and without

exogenous arginine supplementation. (A) Inoculum ratios of all spore pairings used in these race tube experiments; (B) *his-3::hH1-eGFP* (G) *mat A* + *his-3::hH1-mCherry* (R) *mat A* (10BI +74DM) vs. *Δarg-12* (G) *mat A +* (R) *mat A* (131AK + 74DM) spore pairings without supplementation; or (C) with exogenous arginine supplementation, grown for 7 dpi at 30C. Flow cytometry was conducted to analyze the relative percentage of each fluorescently tagged nuclear genotype in asexual spore populations derived from syncytia. Spore pairings in each biological replicate were derived from a single inoculum sample, and the ratios of each partner in the inoculum is shown in the 'Inoculum' graph (Panel A). 'Start' denotes that spores were collected from the opening of the race tube most proximal to the inoculation site on Day 7, and 'End' denotes the spore sample was collected from the portion of the race tube most distal from the site of the inoculation on Day 7. Magenta bars in graphs denote fluorescence signal from homokaryotic spores with the presence of only histone H1-mCherry-tagged nuclei, green bars in graphs denote fluorescence signal from homokaryotic spores with the presence of only histone H1-eGFP-tagged nuclei, and black bars in graphs denote spores with signal from both histone H1-mCherry and histone H1-GFP nuclear tags in the same asexual spore. Statistical analysis was conducted using two-way ANOVA followed by Tukeys HSD post hoc tests. *=$p<0.05$; ** $p<0.01$; ***=$p<0.001$; ****= $p<0.0001$; ns= not significant. N=4. Error Bars = SEM

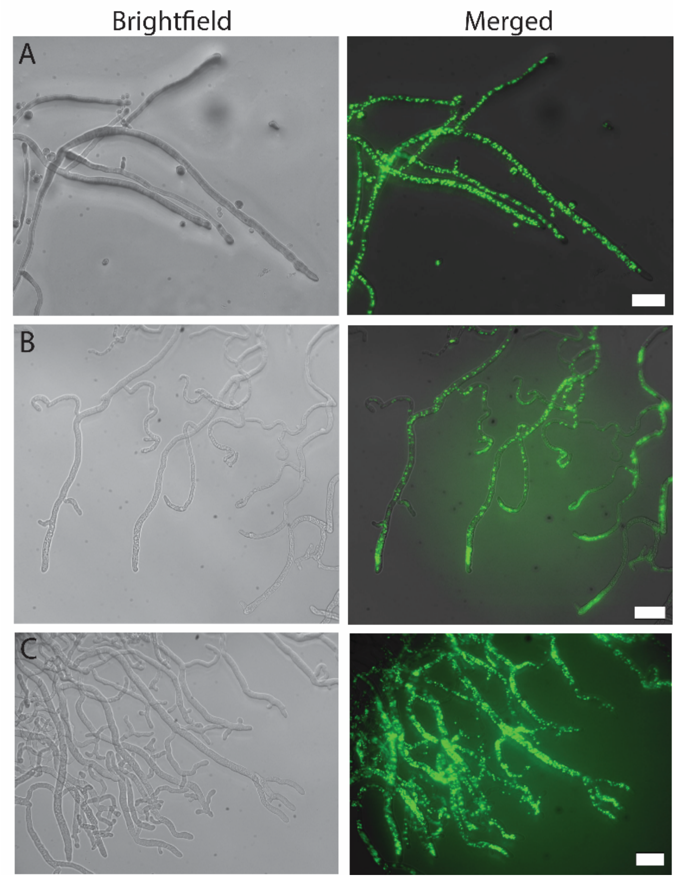

Figure S5. 'Ropy' phenotype of *Δro-3* and *Δro-10* mature hyphae. (A) Normal morphology and nuclear spacing of WT *his-3::hH1-eGFP* (G) *mat A* (93DR) mature hyphae. (B) 'ropy' phenotype and aberrant nuclear spacing of mature *Δro-3* (G) *mat A* (71AO) hyphae. (C) 'ropy' phenotype and aberrant nuclear spacing of mature *Δro-10* (G) *mat A* (72AO) hyphae grown at 30C for 8hpi on solid VMM agar, starting

from a mycelial plug. Representative images shown from two independent experiments. Scale bar = 30 μm.

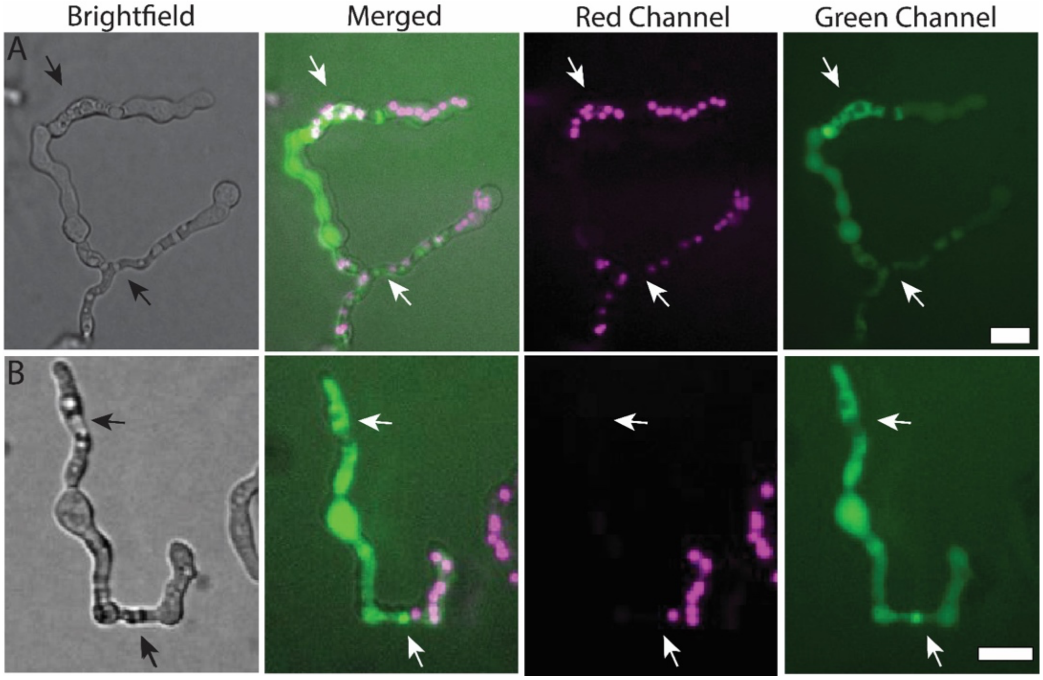

Figure S6. Phenotype of incompatible *rcd-1* germlings of the same and different mating type. (A) Micrographs of germling fusion events between strains of identical mating type, but with different *rcd-1* allelic variants (*csr-1::rcd-1-2 his-3::cytoplasmic-eGFP (G) mat A* + *rcd-1-1 his-3::hH1-mCherry mat A (R)* (5AB + 74DM). (B) Micrographs of germling fusion events between strains of opposite mating type and with different *rcd-1* allelic variants (*rcd-1-2* (G) *mat A* + *rcd-1-1* (R) *mat a* (5AB + 74ED) germlings grown from spores at 30C for approximately 6 hpi in liquid VMM on coverslips. Red fluorescence channel false-colored magenta and overlay of green + magenta may appear white in 'Merged' images. Representative images from two independent experiments shown. Black and white arrows denote regions of fused hyphal compartments where vacuolization has occurred post-fusion. Scale Bar = 10 μm.

References

Detomasi, T. C., A. M. Rico-Ramirez, R. I. Sayler, A. P. Goncalves, M. A. Marletta *et al.*, 2022 A moonlighting function of a chitin polysaccharide monooxygenase, CWR-1, in *Neurospora crassa* allorecognition. Elife 11.

Goncalves, A. P., J. Heller, E. A. Span, G. Rosenfield, H. P. Do *et al.*, 2019 Allorecognition upon fungal cell-cell contact determines social cooperation and impacts the acquisition of multicellularity. Curr Biol 29**:** 3006-3017 e3003.

Ma, L., B. Song, T. Curran, N. Phong, E. Dressaire *et al.*, 2016 Defining individual size in the model filamentous fungus *Neurospora crassa*. Proc Biol Sci 283**:** 20152470.
